# Clinical Application of Deep Learning for Spine MRI Interpretation: A Multicenter Evaluation of Artificial-Intelligence-Assisted versus Manual Reading on Diagnostic Agreement with the Reference Standard

**DOI:** 10.34133/research.1145

**Published:** 2026-02-19

**Authors:** Xing Cheng, Maoping Zhang, Zhenxiao Ren, Tang Tang, Xiaolin Meng, Zhong Huang, Hongwei Bran Li, Weiguo Li, Qiuchan Yan, Haixiong Chen, Jie Jia, Ce Wang, Cheng Li, Chunshan Yang, Guifeng Shi, Guohua Li, Kaixin Zeng, Wei Chen, Haoxuan Gao, Xiaobo Wang, Xin Zheng, Yang Wang

**Affiliations:** ^1^ Department of Spine Surgery, Guangdong Provincial People’s Hospital, Guangdong Academy of Medical Sciences, Southern Medical University, Guangzhou, Guangdong 510080, China.; ^2^ Department of Radiology, Zhujiang Hospital of Southern Medical University, Guangzhou 510280, China.; ^3^ Faculty of Natural Sciences, RWTH Aachen University, 52074 Aachen, Germany.; ^4^ Laboratorio de Regeneración Neuronal, Hospital Nacional de Parapléjicos/IDISCAM, 45071 Toledo, Spain.; ^5^ Department of Spine Surgery, The First Affiliated Hospital, Sun Yat-sen University, Guangzhou 510000, China.; ^6^ Department of Radiology, Nanjing Drum Tower Hospital, The Affiliated Hospital of Nanjing University Medical School, Nanjing 210008, China.; ^7^ Healthcare Advanced Algorithm Department of HSW BU, Shanghai United Imaging Healthcare Co., Ltd, Shanghai 201800, China.; ^8^ School of Electronic Information and Electrical Engineering, Shanghai Jiao Tong University, Shanghai 200240, China.; ^9^ Institute of Neuroanatomy and Cell Biology, Hannover Medical School (MHH), 30625 Hannover, Germany.; ^10^ Department of Diagnostic Radiology, National University of Singapore, 119077 Singapore.; ^11^ Department of Orthopaedics and Traumatology, United Christian Hospital, Hong Kong SAR 999077, China.; ^12^ Department of Radiology, Shunde Hospital of Southern Medical University (Shunde First People’s Hospital), Foshan 528300, China.; ^13^ School of Information Science and Technology, Fudan University, Shanghai 200438, China.; ^14^ Department of Radiology, The First Affiliated Hospital of Qiqihar Medical University, Qiqihar 161041, China.; ^15^ The Second Clinical Medical College of Southern Medical University, Guangzhou 510515, China.; ^16^ Department of Spine Surgery, Nanfang Hospital of Southern Medical University, Guangzhou 510515, China.; ^17^ Department of Orthopedics, The First Affiliated Hospital, Zhejiang University School of Medicine, Hangzhou 310003, China.; ^18^ Department of Radiology, Shanghai Tenth People’s Hospital, Tongji University School of Medicine, Shanghai 20072, China.

## Abstract

Lumbar spine diseases substantially impact the patients’ quality of life, necessitating accurate and efficient diagnostic tools. This study presents Lumbar VNet Pro (LVP), the first real-time artificial-intelligence (AI)-assisted system embedded within MRI hardware for lumbar spine analysis, integrating deep learning with MRI. LVP was trained on 2,453 MRI datasets and validated both internally and externally across multiple centers. During the training (1,848 MRI datasets) and validation (605 MRI datasets), LVP exhibited outstanding performance in localization (Dice = 0.93), segmentation (Dice = 0.92), labeling (identification rate = 0.90), and timeliness (average inference time = 1.1 s). Following the successful construction of LVP, we conducted comprehensive testing through both internal and external multicenter evaluations. Internal testing involving 100 patients indicated that the recognition accuracy of LVP was as high as 100%, and the consistency between the LVP assessment and the manual assessment using the gold standard reached 97%. In external testing involving 1,522 patients, LVP’s diagnostic performance was compared to those of manual and human–machine-assisted methods. The AI-assisted approaches demonstrated better performance across multiple spinal pathologies, including lumbar disc herniation, spinal canal stenosis, and lateral recess stenosis, with area under the receiver operating characteristic curve values >0.95 for deep learning/human–machine approaches and >0.90 for the fully manual approach. The real-time integration of LVP with MRI scanning improved positioning accuracy and reduced interobserver variability, supporting its potential as an adjunct tool for enhancing MRI-based spine diagnostics. However, further studies are warranted to assess its generalizability across diverse clinical settings.

## Introduction

The spine serves as the central functional structure for human movement. Improper movement patterns or injuries resulting from such patterns can directly contribute to the development or worsening of spinal diseases [1]. Among these diseases, lumbar spine conditions are particularly prevalent. Lumbar spine diseases substantially impact the quality of life, making accurate diagnosis and management essential in clinical practice [2–4]. Magnetic resonance imaging (MRI) serves as a cornerstone for lumbar spine evaluation due to its high-resolution imaging and detailed anatomical visualization; however, traditional MRI interpretation is subject to several limitations [5–8]. The reliance on subjective expert assessment and single-dimensional analysis introduces diagnostic variability, with reported error rates exceeding 20% in some cases [9]. Proper MRI positioning is critical for accurate lumbar spine evaluation. According to standardized imaging protocols, axial scans should be reconstructed parallel to the intervertebral disc space to ensure precise assessment of disc morphology; otherwise, degenerative changes in the nucleus pulposus may be obscured [10]. Before image acquisition, localization sequences are used to determine the optimal scanning plane and angle. However, in clinical practice, technologists often rely on subjective experience for positioning [11]. Inexperienced operators may struggle with precise localization, increasing the risk of positioning errors that propagate through subsequent diagnostic interpretations. Such inaccuracies contribute to inconsistencies in spinal alignment measurements, intervertebral disc height assessments, and diagnostic precision, ultimately affecting clinical decision-making. Furthermore, variability in patient positioning during scanning and manual adjustments of localization lines can further impact MRI accuracy, leading to discrepancies in key measurements such as intervertebral disc height and spinal alignment [12–14]. This variability not only contributes to diagnostic uncertainty but also increases patient discomfort and reduces the efficiency of medical resource utilization. Recent advances in deep learning (DL) have introduced promising solutions to these challenges. Unlike traditional methods, DL algorithms can automatically extract and analyze multidimensional features from magnetic resonance (MR) images, enhancing diagnostic objectivity, accuracy, and reproducibility [9,15–22]. Studies have demonstrated that artificial-intelligence (AI)-driven models substantially reduce diagnostic errors by providing comprehensive and quantitative assessments of key spinal structures, including intervertebral discs, nerve roots, and intervertebral foramina [9,16,23–25]. However, despite their potential, most AI models focus on offline image analysis rather than real-time integration with MRI systems. Additionally, further multicenter testing is needed to ensure their robustness across diverse patient populations. To address these limitations, our study introduces Lumbar VNet Pro (LVP), a real-time, fully automated AI model integrated directly with MRI scanning systems. By leveraging DL techniques, LVP enables automated segmentation, positioning optimization, and quantitative analysis of lumbar spine structures, providing immediate feedback during MRI acquisition. This real-time integration enhances diagnostic precision, reduces interobserver variability, and streamlines clinical workflows. Through multicenter testing, we aim to establish LVP as a clinically reliable tool for improving the accuracy, efficiency, and consistency of lumbar spine MRI analysis, ultimately advancing personalized patient care and shaping the future of medical imaging research. Unlike conventional DL models that operate offline after MRI acquisition, the LVP system was developed to achieve real-time integration with MRI scanners. The rationale for selecting a V-Net backbone lies in its proven stability, moderate parameter size, and fast inference speed, which meet the strict latency constraints (<1.5 s) required for on-scanner deployment. Furthermore, the architecture was customized by adapting the convolutional kernel configuration and adding a parameter-estimation head, enabling automatic determination of sagittal/coronal scan centers and saturation-band placement. These capabilities allow dynamic adjustment of scanning parameters during image acquisition. Consequently, the novelty of LVP resides at the system level, representing the first fully automated, device-embedded AI framework for spine MRI positioning and acquisition guidance, bridging the gap between algorithmic intelligence and clinical workflow. This study does not aim to benchmark multiple network architectures but to assess the clinical effectiveness of embedding a robust deep learning model into MRI workflows. The LVP system was developed to achieve real-time AI–MRI interaction, ensuring efficiency, reproducibility, and reduced operator dependence in clinical practice. To our knowledge, this work represents the first large-scale, multicenter clinical testing of a fully MRI-embedded AI system, marking a pivotal step toward the real-world translation of AI in radiology.

## Results

### Patients and study design

**Fig. 1.**
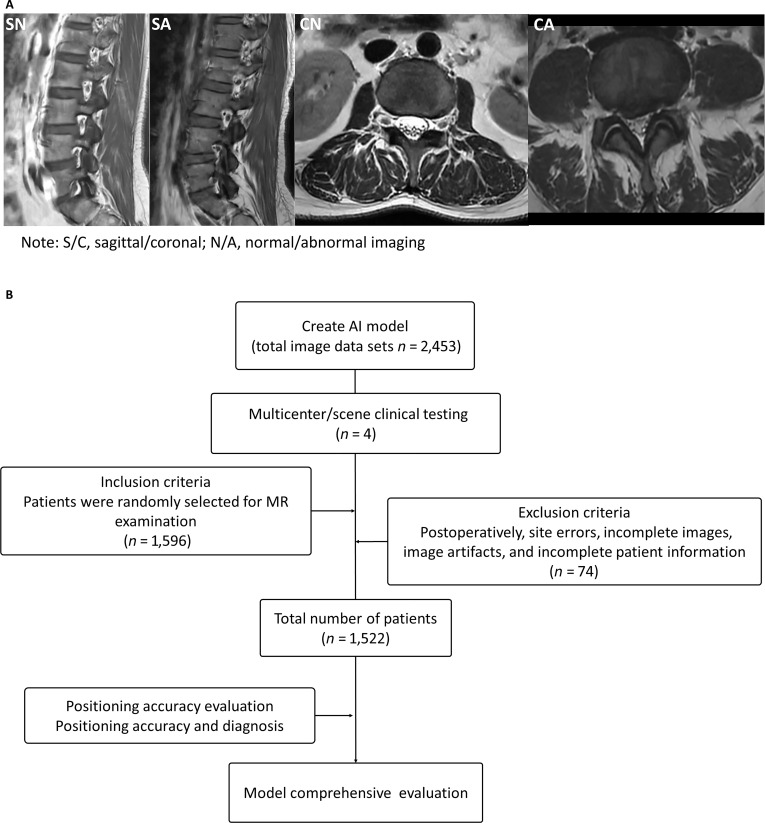
Examples of clinical magnetic resonance (MR) scans that can lead to a misdiagnosis and the workflow diagram. The location design and final diagnosis in clinical MR scans are influenced by the patient’s individual anatomical variations and differences in body positioning during the imaging procedure. (A) The accuracy of the localization was evaluated using sagittal plane parameters (offset angle, lateral foramen vertical diameter, and disc vertical diameter) and cross-plane parameters (psoas area and facet joint angle). (B) The workflow diagram of the entire experimental design outlines the process of multicenter clinical validation of the Lumbar VNet Pro (LVP) model. The testing involved a total of 1,522 patients from 4 centers, and the LVP model underwent further evaluation based on location accuracy and diagnosis rate. AI, artificial intelligence.

**Fig. 2.**
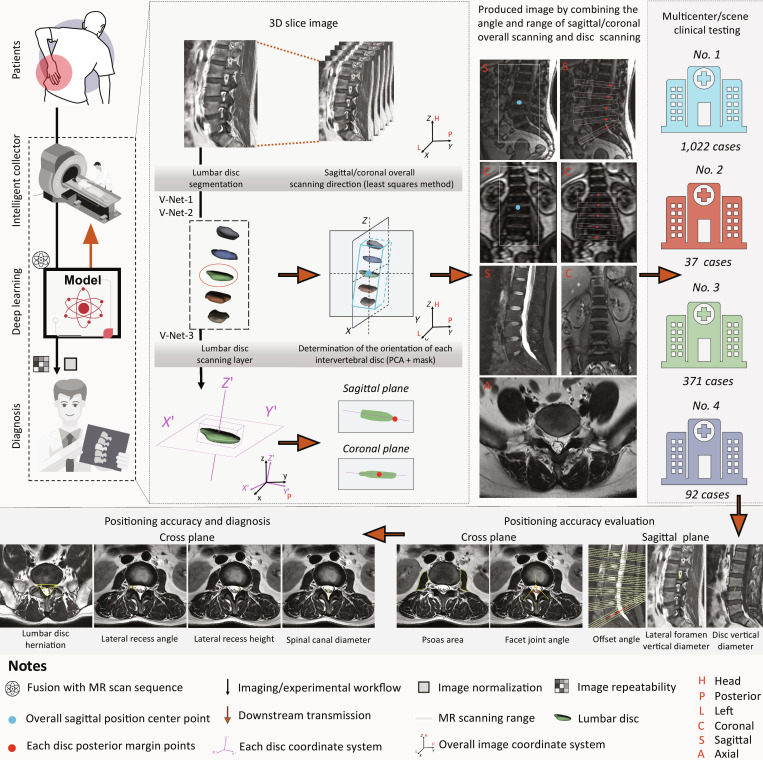
A flowchart of the learning process from the training and testing phase to clinical testing. The MR images were acquired after the patient’s hospital for an MR examination. The obtained MR images are trained and tuned based on the V-Net network for LVP model positioning, segmentation and labeling. The LVP model can combine the angles and ranges of sagittal/coronal holistic scans and disc scans to generate images. Subsequently, an external clinical testing was conducted in a multicenter study involving 1,522 patients. Initially, the accuracy of the model’s localization was evaluated using sagittal plane parameters (offset angle, lateral foramen vertical diameter, and disc vertical diameter) and transverse plane parameters (psoas area and facet joint angle). Following this, the model’s diagnostic detection rates for lumbar disc herniation, spinal canal stenosis, and lateral recess stenosis were assessed. 3D, 3-dimensional; PCA, principal component analysis.

**Fig. 3.**
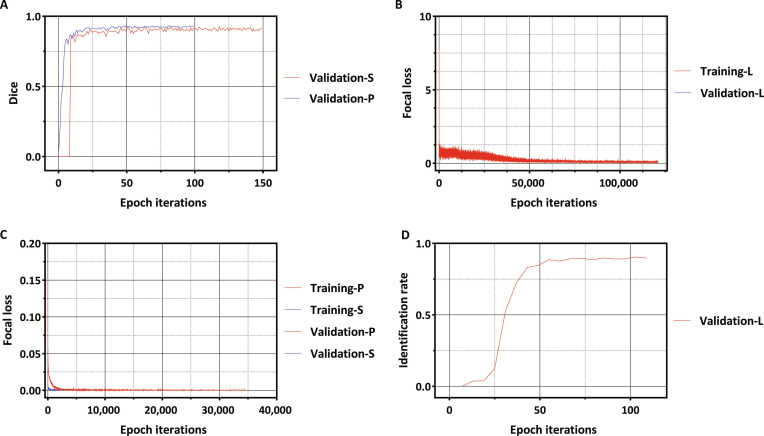
Plot showing the performance of the LVP model during the training and validation phases. The model development process consisted of 2 phases: the training phase and validation phase. During the training phase, the model’s ability to identify and segment intervertebral discs was developed using a training set. The validation set was used to debug and optimize the model until it reached its optimal state. (The model achieved an optimal balance between generalization capability and fitting accuracy.) Debugging results were evaluated through the loss curve and Dice curve. The model was considered qualified when it achieves convergence in the loss curve and a Dice value greater than 0.9. (A) Validation Dice diagram for positioning and disc segmentation module. (B) Training and validation loss diagram of the label module. (C) Training and validation loss diagram of the positioning and disc segmentation modules. (D) Validation identification rate curve of the label module (P, positioning/location module; S, disc segmentation module; L, disc label module. Identification rate: the identification rate was defined as follows: an intervertebral disc was considered correctly identified if the closest centroid in the expert annotation corresponded to the correct disc and the localization error in each 3D direction was less than 6 mm. The identification rate was calculated as the proportion of correctly identified intervertebral discs among all intervertebral discs.

Our study utilized MRI scans from 1,522 adult patients at 4 medical institutions: Gulou Hospital, Qiqihar Medical College, Zhujiang Hospital, and Shunde Medical College, with imaging data collected between 2021 January 1 and 2023 December 31. The design of scan location and final diagnosis in clinical MRI scans is influenced by variations in individual patient anatomy, differences in body position during imaging, and the level of radiologist involvement. The traditional scanning process is hindered by numerous factors, resulting in prolonged and inaccurate positioning time. Hence, an efficient localization method is proposed to substantially enhance clinical work efficiency. We gathered a substantial number of MRI scans and annotated them, subsequently utilizing DL techniques to train and optimize the model until achieving satisfactory results on segmentation accuracy. In the validation set, the model was considered qualified when it achieves convergence in the segmentation accuracy greater than 0.9. Testing was then conducted on a distinct dataset, followed by multicenter verification to further assess the functionality of the model (Figs. 1 to 3 and Tables S1 and S2).

### Model training and validation

The LVP system was first trained on the training set and then proceeded to the validation phase upon the completion of training. During the verification phase, 100 sets of data were meticulously examined by employing comparative models and analyzed by 2 experienced radiologists. Internal testing indicated that the recognition accuracy (region-level labeling accuracy) of the model was as high as 100%, and the consistency between the LVP assessment and the manual assessment using the gold standard reached 97% (Fig. 3 and Tables S3 to S11). The results demonstrated that following training and validation, the LVP system exhibited a commendable level of accuracy. In addition, we confirmed that there were no statistically significant differences in age and gender distribution among the 3 groups. Furthermore, we compared the validation performance of V-Net and a strong segmentation baseline—nnU-Net [26]—across 149 paired test cases using identical training data. Segmentation performance was comparable between the 2 methods, with no statistically significant difference observed (Wilcoxon signed-rank test, *P* = 0.389; Fig. S1).

### Positioning accuracy evaluation among the DL group, HM group, and FM group

**Fig. 4.**
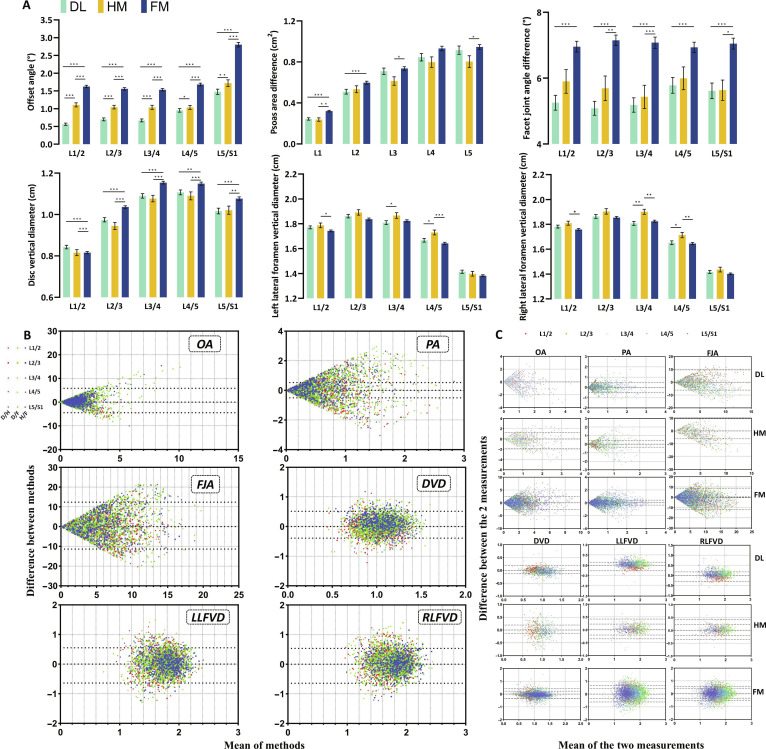
Positioning accuracy evaluation. In multicenter testing, the accuracy of sagittal and coronal orientations was assessed by quantifying the offset angle, disc height, foramen height, psoas area, and facet joint angle in different experimental groups. The smaller the measured values of these parameters, the higher the positioning accuracy. (A) Measured values across different groups. For the offset angle, values consistently followed the order DL < HM < FM. For the psoas area, the FM group consistently exhibited the highest measured values across all groups and spinal segments. For the facet joint angle, values also followed the order DL < HM < FM. For the disc vertical diameter (disc height), DL showed higher values than FM only at the L1/2 segment, whereas FM exhibited the highest measured values in the remaining 4 segments. For the left/right lateral foramen vertical diameter, the FM group showed the minimum measured value in 8 of the 10 measurement groups, whereas the DL group showed the minimum value in the remaining 2 groups. For DL and HM, DL values were lower than HM values in 9 of the 10 measurement groups. (B) Bland–Altman plots were used to assess consistency and agreement among groups for OA, PA, FJA, DVD, and LFVD (D/H, DL vs. HM; D/F, DL vs. FM; H/F, HM vs. FM). The limits of agreement are indicated by dotted lines. Except for the PA measurements, the agreement among different methods was acceptable. Although more data points fell outside the limits of agreement for PA, this may be attributed to the relatively larger measurement error associated with PA. Overall, these results indicate that the DL, HM, and FM methods are interchangeable. (C) Bland–Altman plots were used to assess interobserver consistency between 2 radiologists; the limits of agreement are indicated by dotted lines. Interobserver agreement was acceptable. (*P < 0.05, **P < 0.01, and ***P < 0.001.) DL, deep learning group; HM, human–machine group; FM, fully manual group; OA, offset angle; PA, psoas area; FJA, facet joint angle; DVD, disc vertical diameter; LLFVD/RLFVD, left/right lateral foramen vertical diameter.

We evaluated the accuracy of sagittal and coronal plane positioning by quantifying the offset angle, disc height, foramen height, psoas area, and facet joint angle across various experimental groups (Fig. 4). The patients were divided into the DL group, human–machine (HM) group, and fully manual (FM) group according to different measurement methods.

### Sagittal plane positioning accuracy evaluation

To evaluate the sagittal plane positioning accuracy among the 3 groups, we conducted measurements and assessments of intervertebral disc heights and foraminal heights. The magnitudes of the measured values of disc and foraminal heights were influenced by the choice of the sagittal plane. The measurement yields the minimum value when the optimal sagittal plane is selected. As a result, smaller measured values of these parameters indicated higher precision in the corresponding positioning method. In terms of intervertebral disc height, measurements across all segments exhibited a pattern of HM and DL < FM with significant statistical differences between DL and FM and between HM and FM (*P* < 0.01) but no statistical difference between DL and HM (Fig. 4A). Among the 10 sets of bilateral foraminal height data from the 5 segments, the relative frequencies of the minimum value were as follows: FM, 7/10, and DL, 3/10. There was no consistent statistical difference among the 3 groups, which may be attributed to positional factors during imaging acquisition as well as gender distribution within each group. These results suggested that in sagittal plane positioning, both the DL group and HM group demonstrate superior positioning accuracy compared to the FM group.

### Cross-plane positioning accuracy evaluation

To evaluate the cross-sectional positioning accuracy among the 3 groups, we measured and evaluated the offset angle psoas area and the facet joint angle. When the offset angle and the difference in the measurement values of the facet joint angle and the psoas area was smaller, this indicated a higher cross-sectional positioning accuracy for the corresponding method. In other words, we selected the optimal cross plane. Across the 5 observed segments, the magnitude relationship of offset angles consistently followed DL < HM < FM. Significant statistical differences (*P* < 0.01) were found between DL and HM, DL and FM, and HM and FM. In the 5 segments observed, the psoas area difference was the largest in the FM group. Among these, there were significant differences between the FM and HM groups at L1/2, L3/4, and L5/S1 (*P* < 0.05) and between the FM and DL groups at L2/3 (*P* < 0.0001). There were no statistically significant differences among the 3 groups at L4/5 (Fig. 4A). This indicated that the combination of the LVP system and manual intervention had better results in evaluating cross-sectional positioning accuracy through the psoas area. In all 5 segments, the measurement values of the facet joint angle were DL < FM. There was always a significant statistical difference between DL and FM (*P* < 0.01). Furthermore, a significant statistical difference was observed between HM and FM (HM < FM) at L2/3, L3/4, and L5/S1 (*P* < 0.05). However, the performance of DL and FM in measuring the facet joint angle was similar (*P* > 0.15) (Fig. 4A). The performance of DL in evaluating the facet joint angle was better. The above results showed that the DL group and the HM group have better cross-sectional positioning accuracy than the FM group.

### Consistency evaluation

Bland–Altman analysis revealed marked variability in consistency across different measurement methods (DL, HM, and FM) and anatomical metrics (Fig. 4B and Table S11). For linear measurements such as vertebral canal diameter and disc height, DL demonstrated high consistency with both HM and FM, with biases close to zero and narrow limits of agreement (LoAs) ranging from ±0.5 to 0.6 units. This underscores the robustness of DL in standardized, boundary-defined measurements, where automation minimizes human error and ensures reproducibility. In contrast, for complex metrics such as facet joint angle and lateral recess height/angle, DL showed pronounced inconsistency with FM, with LoA ranges as wide as ±4.0 to 14.0 units. This variability is largely attributed to the high operator dependency and subjectivity of FM measurements, particularly in complex anatomical regions where manual reference point selection can vary substantially. HM, which combines AI with manual intervention, exhibited intermediate consistency, performing better than FM but slightly worse than DL, suggesting that while AI assistance reduces variability, manual intervention in complex regions can still introduce some degree of inconsistency. These findings highlight the strengths of DL in achieving high measurement consistency, particularly in linear metrics, while also emphasizing the challenges of manual methods in complex anatomical assessments. The results underscore the potential of DL to improve diagnostic accuracy and reduce variability in clinical practice, particularly when combined with selective human oversight in complex cases. We also evaluated the consistency of the measurements made by the 2 radiologists using Bland–Altman analysis. The results indicated that the consistency of the measurements made by the 2 doctors was acceptable (Fig. 4C).

### Effect of precise patient positioning on diagnostic accuracy

**Fig. 5.**
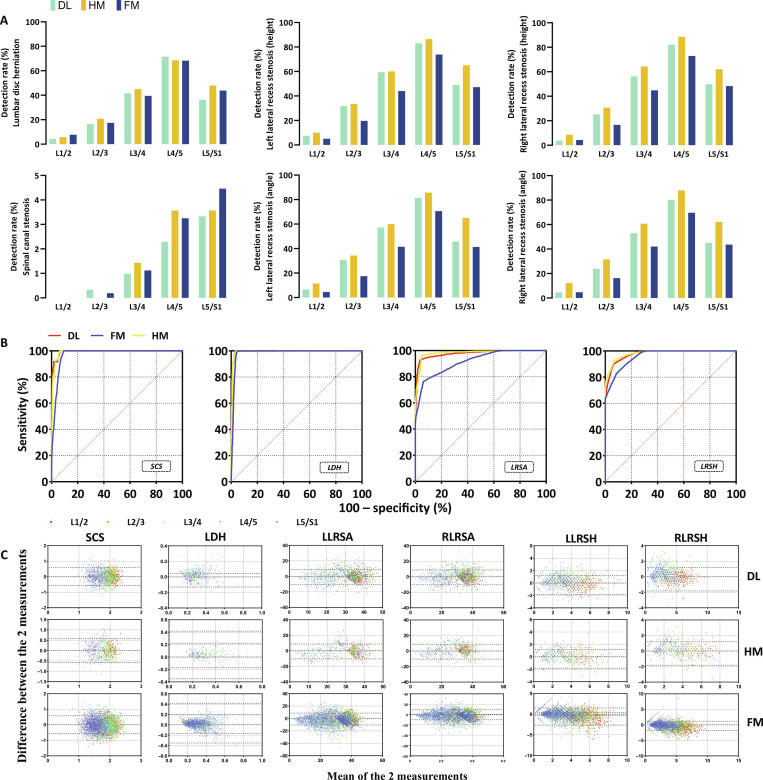
Role of accurate patient positioning in ensuring diagnostic precision. The diagnostic detection rates of lumbar disc herniation, spinal canal stenosis, and lateral recess stenosis (LRS) were evaluated and compared across different methods. Lumbar disc herniation (LDH) was evaluated using the disc herniation rate and Michigan State University (MSU) classification. A spinal canal diameter <10 mm indicates spinal canal stenosis (SCS), and a lateral recess height <3 mm or a lateral recess angle <30° indicates LRS. (A) Out of the 10 diagnostic comparisons between LDH and SCS, FM exhibited the highest diagnostic detection rate only twice out of 10 times (2/10). The diagnosis rate of LRS in DL and HM was higher compared to that in the FM group (20/20). (B) The receiver operating characteristic (ROC) curve shows that in the diseases of LDH, SCS, and LRS involved in the identification, DL, HM, and FM all have very superior diagnostic performance, but the diagnostic performance of DL and HM is even more excellent than that of FM. (C) The Bland–Altman plot was used to assess the consistency of the same measurement values measured by 2 radiologists; the limits of agreement are indicated by dotted lines. The consistency of the 2 radiologists’ measurements was acceptable. LLRSA/RLRSA, left/right lateral recess stenosis (angle); LLRSH/RLRSH, left/right lateral recess stenosis (height); DL, deep learning group; HM, human–machine group; FM, fully manual group.

The subsequent step involved assessing the detection rates of lumbar disc herniation, spinal canal stenosis, and lateral recess stenosis utilizing various methodologies. Lumbar disc herniation was assessed using the disc herniation ratio and the Michigan State University (MSU) classification. A spinal canal diameter <10 mm indicated spinal canal stenosis, while a lateral recess height <3 mm or a lateral recess angle <30° indicated lateral recess stenosis. In the 5 segments examined, the diagnostic detection rates of all segments were compared and the highest detection rate was selected. Among the 5 comparisons of lumbar disc herniations, the highest diagnostic detection rates of HM (3/5) were as follows: L2/3, 20.71%; L3/4, 45%; and L5/S1, 47.86%. In the 5 comparisons, DL (L4/5, 71.57%) and FM (L1/2, 7.71%) were both 1/5. In the comparison of spinal canal diameters, the diagnostic detection rates of HM were the highest (2/5), which were as follows: L3/4, 1.43%, and L4/5, 3.57%. Among them, DL (L2/3, 0.33%) and FM (L5/S1, 4.46%) were both 1/5. In contrast, for lateral recess, a total of 20 comparisons were conducted on the height and angle of the lateral recess on both sides, and the results showed that the diagnostic detection rate of the HM group was the highest in the 20 comparison experiments (20/20). Among them, the highest detection rate of a single segment can be as high as 88.57% (Fig. 5). HM has a higher detection effect on disease. Receiver operating characteristic (ROC) analysis was conducted to evaluate the diagnostic efficacy of DL, HM, and FM for the 3 diseases. For lumbar disc herniation, high diagnostic performance was achieved across all 3 methods, with area under the ROC curve (AUC) values of 0.9942 for DL, 0.9932 for HM, and 0.9832 for FM (all *P* < 0.0001). For spinal canal stenosis, the DL, HM, and FM methods demonstrated strong diagnostic performance, with AUC values of 0.9928, 0.9905, and 0.9719, respectively (all *P* < 0.0001). For lateral recess stenosis assessed based on height measurements, the AUC values were 0.9773 for DL, 0.9817 for HM, and 0.9603 for FM (all *P* < 0.0001). When lateral recess stenosis was assessed based on angle measurements, the corresponding AUC values were 0.9775 for DL, 0.9822 for HM, and 0.9176 for FM (all *P* < 0.0001) (Fig. 5B). The results showed that DL, HM, and FM all have good diagnostic efficacy, but DL and HM have better performance compared to FM. In addition, Bland–Altman analysis was used to evaluate the consistency of the measurement results of the 2 radiologists. The results indicated that the consistency of the measurement values of the 2 doctors was acceptable (Fig. 5C).

## Discussion

The development and application of AI in clinical settings have markedly enhanced the efficiency of radiologists. However, AI has not yet been widely integrated into the most upstream stage of medical imaging—the acquisition process—where substantial variability in operator proficiency remains. To address this gap, we developed LVP, which combines DL and MRI radiomics in a real-time, device-embedded workflow. The LVP system represents the first real-time AI framework that closes the loop between image acquisition and AI-based analysis. Its novelty lies in integrating deep learning modules directly into the MRI scanning workflow, enabling automatic positioning, plane optimization, and parameter generation without human intervention.

While LVP adopts a V-Net-based backbone, its primary contribution lies in system-level integration rather than architectural novelty. To contextualize its algorithmic performance, we directly compared the V-Net backbone used in LVP with the state-of-the-art nnU-Net under identical data splits and evaluation protocols. Across 149 paired test cases, the 2 methods demonstrated comparable segmentation performance, with no statistically significant difference observed (Wilcoxon signed-rank test, *P* = 0.389). This finding indicates that the V-Net backbone employed in LVP achieves performance statistically equivalent to that of a contemporary segmentation framework.

Architecturally, nnU-Net concentrates model capacity within a single auto-configured network and relies on patch-based inference, whereas LVP distributes a comparable level of capacity across multiple task-specific V-Net modules executed sequentially. Despite these differences, segmentation accuracy remained similar. Accordingly, the choice of V-Net in LVP was driven by deterministic inference latency, predictable memory usage, and deployment maturity, which are critical for real-time, device-embedded MRI applications. Importantly, this study was not designed as an algorithmic benchmark across network architectures, but rather as a clinical evaluation of AI-assisted operation during MRI acquisition, consistent with its translational focus. Accordingly, the DL/HM/FM comparison reflects different levels of human–AI collaboration in real-world practice.

In this study, the findings indicated that in the analysis of lumbar spine MR images, the fully automated AI group exhibited superior accuracy across multiple anatomical structures when compared to the HM coupled and FM groups. During the phase of precise positional assessment, a lower recorded value for pertinent parameters signifies an elevated degree of precision in localization. In the comparison of offset angle, psoas area difference, and facet joint angle difference, the FM measurement accounted for 100% of the maximum. Both the DL and HM groups demonstrated smaller magnitudes compared to the FM group, and the smallest measured value of DL was 80%, and the difference between groups was obvious. The integration of AI technology facilitates generation of more accurate positioning lines. The accuracy of the results can be enhanced by calculating the difference value, which eliminates differences caused by anatomical and pathological factors when comparing both sides of the body. In the comparison of intervertebral disc height measurements, the FM group exhibited the highest measured values in 80% of cases, whereas the DL group exhibited the lowest measured values in 80% of cases, indicating a statistically significant difference between groups. This suggests that the AI-assisted measurement approach may provide more precise and consistent disc height evaluations compared to FM measurements. It was noteworthy that intervertebral disc height is closely correlated with patients’ age and degree of degeneration, and variations in patient demographics can also influence measurement outcomes [27–29]. In terms of lateral foramen height comparison, the proportion of HM with the largest measurement was 90%, and the proportion of FM with the smallest measurement was 80%. Within this cohort, no significant differences were observed among the 3 groups, which could potentially be attributed to specific anatomical factors influencing foraminal dimensions. Factors such as age, gender, anatomical variations, and posture have been found to influence foraminal height [30–32]. Notably, men tend to have larger foramina compared to women and there is a decrease in size associated with aging and lumbar degeneration [30,32]. Due to sample randomness, it was not possible to achieve identical age distribution, sex ratio, or disease status across different groups; this may account for these findings. In the stage of disease detection and evaluation, a higher detection rate leads to better outcomes. When comparing lumbar disc herniation, spinal canal stenosis, and lateral recess stenosis, 83.3% of patients with HM achieved the highest detection rate compared to only 6.7% of DL patients. Therefore, the HM group exhibited a superior diagnostic detection rate.

The poor performance of the FM group may be related to factors such as the radiologist’s years of experience, skill level, expertise, operational habits, and adherence to standardized procedures. Additionally, individual subjective awareness and work status may also influence measurement results [15,16]. Our algorithm can provide a more objective, precise, and consistent approach in diagnostic applications. However, in terms of disease detection performance, while the fully automatic AI group is clearly superior to the FM group, a gap still remains compared with its performance when coupled with HM interaction. This could be attributed to situations where physicians can correct images that are not standard due to complex imaging or nonstandard positioning during manual intervention based on rich experience—situations that AI finds difficult to accurately assess [15]. This suggested that additional human intervention in AI can greatly improve disease detection rates and potentially have a substantial positive impact on actual clinical practice in the future. This groundbreaking research provides a solid foundation for the application of AI and DL to imaging assessment in lumbar spine diseases, offering robust support for future research and clinical applications. In addition, the Bland–Altman analysis highlights the superior consistency of DL in linear measurements such as vertebral canal diameter and disc height, where automation minimizes human error and ensures reproducibility. However, the wider LoAs observed for complex metrics like facet joint angle and lateral recess height/angle underscore the challenges of manual methods in complex anatomical assessments. These inconsistencies are likely due to the high operator dependency and subjectivity inherent in FM measurements, particularly in regions requiring precise landmark identification. The intermediate performance of HM suggests that while AI assistance can reduce variability, manual intervention in complex regions may still introduce some degree of inconsistency. These findings emphasize the potential of DL to enhance diagnostic accuracy and reduce variability in clinical practice, particularly when combined with selective human oversight in complex cases. Future studies should focus on optimizing hybrid approaches and expanding datasets to include diverse anatomical variations, further validating the robustness of AI-driven methods across different clinical scenarios.

The application of MRI AI and automatic segmentation algorithms in the medical field is multifaceted and has made substantial advancements. MRI plays a crucial role in the diagnosis and treatment of brain diseases (brain tumors, cerebral hemorrhages, and strokes), cardiovascular diseases (coronary artery disease and myocardial infarction), musculoskeletal and joint diseases (fractures, arthritis, and synovial sarcomas), and so on [33–37]. These algorithms assist medical professionals in making more precise diagnoses and tracking disease progression. In lumbar diseases, previous research has predominantly concentrated on developing automated segmentation algorithms to accurately identify and segment structures such as intervertebral discs and vertebrae within MRI [9,16]. These algorithms are often based on traditional image processing techniques like edge detection and region growing. However, conventional methods may exhibit limitations in handling complex image variations and noise [16]. In recent years, DL techniques, particularly convolutional neural networks, have made substantial breakthroughs in MRI analysis [18,38,39]. Researchers have designed various DL architectures for feature learning and segmentation of images. These networks can automatically learn high-level image features, thereby enhancing segmentation accuracy and robustness. Some studies have applied 2-dimensional and 3-dimensional (3D) convolutional network structures to MRI segmentation tasks to capture more comprehensive spatial information [15,16,40]. Besides segmentation, AI technologies have also been used for MRI registration and reconstruction. Registration techniques align MRI acquired at different time points or with different modalities for comparative purposes. Reconstruction methods focus on recovering high-quality MR images from limited sample data to reduce scan time and improve image quality [16,18]. Here, we integrate an intelligent localization algorithm with DL techniques, enabling comprehensive segmentation and label localization of spinal localization images, surpassing the scope of intervertebral disc segmentation. This comprehensive approach enhances accuracy. This proposed approach is the first to integrate AI with scanning equipment, as well as being pioneering to validate model performance through a combination of retrospective and prospective studies.

The previous approach solely focused on postprocessing the image, neglecting intervention in the most upstream of image acquisition. However, it is crucial to recognize that image acquisition plays a pivotal role in MRI scanning as it substantially impacts subsequent image processing and analysis. In this study, we successfully integrated AI technology with scanning equipment for the first time, enabling more precise and expedited positioning during the scanning stage. Consequently, this breakthrough resulted in faster and more accurate generation of MRI images. Notably, our clinical observations reveal that a fully LVP-guided MRI operation can be completed within a mere 10 to 15 s compared to traditional MRI practices, which are typically over 10 times longer at approximately 2- to 3-min duration. The 10 to 15 s encompass both the machine’s response time and human adjustment time (i.e., the confirmation period by operators). As shown in our real-world application scenario video, when using only the LVP system for positioning, the time required becomes much shorter—nearly real-time localization. This remarkable improvement is primarily attributed to automated scanning procedures devoid of human intervention, effectively streamlining the positioning process (Movie S1). Furthermore, the introduction of our intelligent localization algorithm provides automatic generation of optimal sagittal and coronal view images. This not only enhances image visualization but also improves diagnostic accuracy. In addition, our research attains unprecedented precision and comprehensiveness, particularly in assessing various lumbar anatomical structures like intervertebral disc heights, lumbar muscle cross-sectional areas, and facet joint angles. These improvements offer clinicians more precise information, enhancing clinical decision-making. Moreover, our results undergo testing in diverse clinical scenarios, including the DL-MRI group, HM coupling group, and FM group. This multiscenario testing enhances the versatility and practicality of our algorithm, making it adaptable to various clinical requirements.

Although LVP is built upon a V-Net-based black-box model and does not explicitly expose internal decision logic, clinical trust is primarily established at the system level rather than through intrinsic model interpretability. LVP is deliberately designed as a nondiagnostic, assistive tool for vertebral and intervertebral disc localization during MRI acquisition, with clearly defined task boundaries, full visualization of outputs, and complete human-in-the-loop control. By restricting AI outputs to editable localization guidance rather than diagnostic decisions, potential errors are confined to workflow efficiency and do not directly impact clinical decision-making, thereby mitigating the risks associated with model opacity. Moreover, the consistent and standardized visualization of localization results provides implicit educational value, particularly for less experienced operators. While real-time interpretability is not required for on-scanner deployment, future extensions will explore post hoc interpretability and uncertainty-aware approaches [41,42], as well as conformal prediction to provide distribution-free confidence guarantees under dataset shift [43,44], as complementary, non-real-time tools to further enhance transparency, quality control, and clinician confidence without compromising real-time performance.

While LVP adopts a V-Net backbone, its integration with the scanning parameter calculation module and real-time MRI feedback distinguishes it from conventional offline networks. This combination enables automatic determination of sagittal/coronal planes and saturation-band placement during image acquisition, achieving a functional advance beyond algorithmic segmentation accuracy alone. nnU-Net is also a widely used benchmark model in medical image segmentation, excelling in offline processing and handling complex anatomical structures with high accuracy [26]. In contrast, LVP extends AI applications into real-time clinical settings by integrating directly with MRI equipment, enabling immediate scanning guidance and positioning. This makes LVP particularly suitable for dynamic environments like spinal surgeries. This is to a certain extent in contrast to the nnU-Net + scanning parameter calculation module. Furthermore, the core of this article lies in presenting an automatic positioning framework based on AI models. Under this framework, any outstanding AI model can help achieve the automation of lumbar spine scans while obtaining high-quality MR images, thereby assisting clinicians in making precise diagnoses. Additionally, LVP introduces innovations in comprehensive automation and system integration, allowing for detailed evaluations of lumbar anatomy beyond standard segmentation tasks. In our study, the real-time nature of LVP was assessed through both quantitative inference benchmarking and qualitative empirical observation. Our benchmarking tests indicate an average inference time of 10 to 15 s per scan, which ensures seamless integration into clinical workflows. Additionally, a supplementary movie demonstration is provided to illustrate the immediate generation of diagnostic insights following MRI acquisition, further validating the system’s real-time functionality in a visually intuitive manner. Although LVP demonstrates real-time inference (1 to 1.5 s per case) on an MRI-console-grade graphics processing unit (GPU), its performance is not dependent on specialized or high-end hardware. We evaluated LVP across representative GPU configurations ranging from legacy, cost-sensitive systems (NVIDIA GTX 1060, 6 GB) to more recent mid- to high-end clinical setups (NVIDIA RTX 2060 SUPER, 8 GB) and consistently observed inference times within 1.5 s, meeting standard MRI workflow requirements. These results indicate that LVP can be integrated into existing clinical MRI systems without mandatory hardware upgrades, supporting cost-effective and broad deployment. The favorable computational efficiency is partly attributable to the use of a V-Net-based architecture, which balances segmentation accuracy with runtime efficiency and is well suited to resource-constrained environments. Looking forward, further model lightweighting and alternative deployment strategies, such as centralized or cloud-based inference, may provide additional pathways to enhance scalability and accessibility, particularly in settings with limited local computational resources.

Fully automating MRI scanning and positioning through an AI-embedded system such as LVP has the potential to reshape the professional training structure in radiology. Traditionally, acquiring expertise in lumbar MRI scanning requires extensive manual experience and years of operator training to achieve consistent quality. With LVP, the threshold for mastering high-quality image acquisition can be markedly reduced, as the system performs automated positioning, parameter adjustment, and scan-plane optimization. This may shorten the learning curve for novice technologists and lower institutional training costs. From a clinical workflow perspective, the automation allows human experts to shift their role from manual operation toward supervision and testing of AI-generated outputs, enabling technologists to focus more on quality control and patient management rather than repetitive positioning tasks. In the long term, such systems may facilitate standardization across institutions and improve interoperator consistency while still requiring expert oversight to ensure safety and reliability.

Our study has several limitations. One notable limitation is the generalizability and representativeness of the data. Although we employed multicenter external testing to enhance reliability, the majority of subjects were recruited from selected clinical centers, and the number of cases contributed by different centers was unequal. This imbalance was partly due to the staggered deployment of the United Imaging (UI) MR system across participating hospitals, which influenced case accumulation rates. Additionally, differences in scanner vendors, imaging sequences, and patient positioning techniques may affect the performance of the LVP system across sites. As a result, the dataset may not fully reflect the diversity of the general population or the full range of MRI protocols used in wider clinical practice. To improve generalizability and ensure robust clinical applicability, future research should aim to validate the algorithm across more heterogeneous populations, a broader spectrum of spine pathologies, and different healthcare settings. Secondly, despite rigorous expert labeling and arbitration processes, there may still be interexpert variability in defining ground truth standards for anatomical measurements, which introduces potential uncertainty into algorithm testing. Moreover, the implementation of AI-assisted tools often entails additional costs and workflow adjustments, which may impact adoption. Assessing the cost-effectiveness and integration logistics of LVP is therefore essential for widespread deployment. Finally, we did not conduct stratified analyses based on age or lumbar degeneration severity. Since these clinical factors influence spinal morphology and interpretation, subgroup analyses could help determine whether the LVP system performs consistently across varying stages of degeneration. Future work should address this gap to further support the model’s applicability in diverse patient subgroups.

This study highlights the value of integrating AI and DL into lumbar spine imaging. By overcoming previous limitations and improving the accuracy of anatomical assessment, the proposed approach contributes to the development of clinically integrated imaging. The LVP system provides a practical solution for the diagnosis and treatment of lumbar spine disorders by embedding AI directly into MRI scanners, thereby enabling real-time acquisition guidance, automatic plane optimization, and parameter adjustment. This system-level innovation improves diagnostic accuracy, consistency, and workflow efficiency, effectively linking algorithmic intelligence with imaging hardware. To our knowledge, this work represents the first large-scale, multicenter clinical testing of an AI system fully integrated within MRI hardware, demonstrating the feasibility and clinical potential of real-time AI-assisted MRI acquisition. By reducing operator variability and standardizing image quality across centers, the LVP framework offers a reproducible model for the clinical translation of AI technologies in medical imaging. Further studies are warranted to evaluate its long-term performance across diverse MRI platforms and patient populations and to optimize deployment in routine clinical practice.

## Materials and Methods

### Study design and patients

This study was approved by the Institutional Review Board/Ethics Committee of Zhujiang Hospital of Southern Medical University (Approval No. 2023-KY-290-01). Given the retrospective nature of the study and its reliance on existing MRI scans, written informed consent from patients was not required. The research was conducted in accordance with the principles outlined in the Helsinki Declaration, ensuring strict compliance with ethical guidelines and patient data protection regulations.

The accuracy of scan localization and final diagnosis in lumbar spine MRI is influenced by individual anatomical variations, patient positioning during imaging, and radiologist involvement. Traditional scanning workflows are subject to multiple challenges, often resulting in prolonged acquisition times and positioning inaccuracies (Fig. 1 and Fig. S2). To address these limitations and improve workflow efficiency, we developed an AI-assisted localization system. A substantial dataset of MRI scans was collected and manually annotated, followed by DL-based training and optimization until the model achieved satisfactory performance based on the loss/Dice curve. The model was considered validated when the loss curve converged and the Dice coefficient exceeded 0.9.

Following internal testing, a multicenter evaluation was conducted to further assess the model’s robustness. The testing process involved quantifying key anatomical and diagnostic parameters, including offset angle, lateral foramen vertical diameter, intervertebral disc vertical diameter, psoas muscle cross-sectional area, facet joint angle, and disease detection rates, to verify the accuracy of the LVP system (Figs. 2 and 3).

Our study utilized MRI scans from adult patients at 4 medical institutions: Gulou Hospital, Qiqihar Medical College, Zhujiang Hospital, and Shunde Medical College, with imaging data collected between 2021 January 1 and 2023 December 31. Strict privacy and compliance protocols were adhered to throughout the study. Exclusion criteria included postoperative scans, imaging site errors, incomplete scans, image artifacts, and missing patient information, ensuring a high-quality dataset for model development and validation.

### Datasets and groups

This research utilized multiple datasets for model development and validation, categorized as follows: (a) development dataset, which was used for model training and validation, and (b) validation dataset, which was employed for independent performance evaluation, including both internal and external validation cohorts.

The development dataset comprised 2,453 MRI scans, further divided into 3 distinct training sets (657/467/724) and validation sets (193/148/264) to optimize the spine localization, intervertebral disc segmentation, and vertebral disc labeling modules. The internal testing set included 100 randomly selected lumbar spine MRI scans from the UI MR system, ensuring unbiased model assessment. Notably, given the prevalence of vertebral and disc abnormalities in lumbar spine imaging, the internal testing dataset also included 31 cases of vertebral body abnormalities, 69 cases of normal vertebrae, 22 cases of intervertebral disc abnormalities, and 78 cases of normal discs. This inclusion facilitated a more comprehensive evaluation of the algorithm’s robustness and clinical applicability.

To benchmark the segmentation backbone used in LVP against a contemporary state-of-the-art approach, we performed a direct comparison between the V-Net architecture employed in LVP and nnU-Net using identical data splits and evaluation protocols. nnU-Net was trained with its standard 3D full-resolution auto-configured pipeline without manual tuning. Segmentation performance was evaluated on 149 paired test cases.

The external independent testing dataset consisted of 1,522 lumbar spine MRI scans and was structured into 3 distinct groups for comparative analysis: (a) DL group: This included 306 patients from Gulou Hospital who underwent fully automated lumbar AI MRI scans without manual intervention. The mean age of this group was 48.64 ± 15.36 years, with a male-to-female ratio (M:F) of 121:185. (b) HM group: This included 140 cases from Gulou Hospital, in which AI-assisted scanning was complemented by manual adjustments made by radiologists. The LVP system initially performed automated spine localization and segmentation, generating key scan parameters such as sagittal scan center, coronal scan alignment, and saturation-band positioning. A radiologist then reviewed these parameters and, if necessary, manually refined them before the final scan acquisition. This hybrid workflow—comprising AI preprocessing, radiologist verification, manual parameter refinement, and final scan execution—allowed for an in-depth evaluation of how expert oversight enhances AI-assisted imaging. The mean age of this group was 47.88 ± 17.78 years, with an M:F of 62:78. (c) FM group: This included 1,076 cases from 4 institutions (Gulou Hospital [576], Qiqihar Medical College [371], Zhujiang Hospital [37], and Shunde Medical College [92]). In this group, radiologists followed a conventional manual workflow, independently determining spine localization, slice positioning, and intervertebral disc segmentation without AI assistance. The mean age of this group was 49.08±16.51 years, with an M:F of 455:621.

To ensure the broad applicability of the LVP system, the external testing dataset incorporated MRI scans acquired from multiple manufacturers, including GE, Philips, UI, and Siemens. This multivendor, multicenter testing framework was designed to rigorously assess the generalizability and clinical reliability of the proposed AI model.

### Annotation process and quality control

In this study, we developed the LVP AI model to enhance lumbar spine MRI analysis. Prior to model training, all images in the training and validation datasets were annotated by 2 senior independent radiologists, each with over 10 years of clinical imaging experience. Initially, both radiologists independently annotated the images using the ITK-SNAP software (version 3.8.0, Cognitica, Philadelphia, PA, USA), a widely used open-source medical image segmentation tool. Following the initial annotation, a third expert reviewed and validated the ground truth for each case. Any discrepancies were resolved through a consensus process, ensuring consistency and minimizing human errors.

The annotation process was structured into 3 key components: spine segmentation, intervertebral disc segmentation, and intervertebral disc labeling (Fig. S3). First, the spine mask was delineated to ensure comprehensive coverage of the vertebral bodies and intervertebral discs. Second, the intervertebral disc mask was segmented, precisely defining each disc’s shape and boundaries. Lastly, the central position of each disc was identified and labeled to ensure accurate localization. To maintain annotation quality, rigorous evaluation criteria were established: (a) The spine mask had to precisely align with the spinal contour. (b) The intervertebral disc mask had to accurately reflect disc morphology. (c) The disc labels had to be correctly assigned to their respective anatomical positions.

These procedures were adapted from previously established lumbar spine segmentation methodologies [45]. This standardized annotation framework ensured high-quality, reproducible labels, providing a robust foundation for both model training and validation.

### Algorithm model training, validation, and development

The LVP system conducts segmentation and label localization of intervertebral discs on spinal positioning images. It automatically selects the best display sagittal and coronal images and computes the scanning parameters, including the sagittal scan center and orientation, coronal scan center and orientation, transverse scan center and orientation, saturation bands, disc labels, vertebral body labels, and quality factors (Fig. S4). The intelligent spine localization algorithm based on positioning images comprises 4 major modules: the spine localization module, intervertebral disc segmentation module, intervertebral disc label module, and scanning parameter calculation module.

We expanded the Materials and Methods to specify the input–output of each module. The LVP pipeline ingests 3D MRI localizer volumes (sagittal and coronal). The segmentation head outputs voxel-wise class–probability maps, the localization/label modules output disc centers and vertebral/disc labels, and the parameter-estimation head outputs numerical scan-plane parameters (sagittal/coronal centers and saturation-band positions) used by the scanner. We also report the approximate number of trainable parameters for the backbone and heads (Table S13).

#### Spine localization module

The spine localization algorithm performs a coarse segmentation of the input data, utilizing the minimum bounding rectangle of the segmentation result as the localization outcome for the spine. Subsequent algorithms are executed within the defined spine localization region. We employed a segmentation network based on the V-Net architecture as a prototype. V-Net is a fully convolutional neural network specifically designed for volumetric medical image segmentation, extending the principles of U-Net by incorporating residual connections to enhance feature propagation and gradient flow [15,46–48]. The choice of V-Net over more recent architectures (e.g., nnU-Net or transformer-based networks) was deliberate and driven by engineering considerations for real-time MRI integration. V-Net offers a favorable balance between segmentation accuracy and computational efficiency, allowing inference within 1 to 1.5 s per case on MRI-console-grade GPUs. This latency is critical for embedding the algorithm directly into the scanner workflow. Furthermore, the standard V-Net was customized for the LVP system by (a) optimizing convolutional kernel sizes to match MRI localizer resolutions, (b) modifying the input–output tensor dimensions for 3D localization, and (c) appending an additional parameter-estimation head for automatic calculation of sagittal and coronal scanning planes as well as saturation-band placement. These custom adaptations enable real-time feedback between the AI model and MRI acquisition, which is not feasible with existing offline segmentation networks. The model is well suited for medical imaging applications due to its ability to effectively capture spatial dependencies and maintain high segmentation accuracy, particularly in complex anatomical structures. This network is a typical encoder–decoder structure. During the encoding phase, image features are extracted through convolutional operations, gradually reducing the spatial scale. In the decoding phase, deconvolution operations are employed to gradually recover target details and spatial scale.

The image dataset used for this purpose was collected from the UI Healthcare superconducting MR equipment, consisting of 657 sets of training data and 193 sets for validation data. The spine masks were annotated by qualified personnel and underwent quality assessment by clinical experts, with arbitration for any discrepancies.

The segmentation network was trained and tested using the PyTorch framework. The training hardware environment was a DGX workstation (central processing unit [CPU]: Intel Xeon E5-2698 v4 2.2 GHz, 256 GB of random-access memory [RAM]; NVIDIA GPUs: 4X Tesla V100, 128 GB of video random-access memory [VRAM]). The testing hardware environment was an Intel Core i5-8500 CPU at 3.00 GHz, 16 GB of RAM, and NVIDIA GeForce GTX 1060 with 6 GB of VRAM. The algorithm utilizes the commonly used Dice coefficient for model selection and evaluation. A selected model is considered qualified if it meets 2 evaluation criteria: convergence of the training and validation loss curves and a validation Dice value exceeding 0.9 (Fig. 3).

#### Intervertebral disc segmentation module

The intervertebral disc segmentation module is used to obtain masks for each intervertebral disc. Within the spine localization region, the V-Net segmentation network is employed to segment the intervertebral discs, resulting in segmentation outcomes for all intervertebral discs in the sagittal and coronal localization images. Subsequently, we located the centers of the segmented intervertebral discs. We then utilized 467 training data and 148 validation data from data A for model training, which was conducted over 150 epochs. The final model underwent testing on 100 images, with the gold standard for testing established by 2 experts.

During the training process, we used focal loss and Dice for evaluation. The focal loss formula is expressed as Eqs. (1) and (2):

$$\mathrm{FL}\left(p_{t}\right)=-{\left(1-p_{t}\right)}^{\gamma }\;\text{log}\left(p_{t}\right),$$ (1)

$$p_{t}=\left\{\begin{array}{cc} p & \text{if}\;y_{\text{pred}}=\text{label},\\ 1-p & \text{otherwise}, \end{array}\right.$$ (2)

where $p_{t}$ represents the predicted probability of the target class and *γ* (gamma) is a focusing parameter that adjusts the weighting of hard-to-classify samples. When an example is misclassified (low $p_{t}$), the modulating factor is near 1, and the loss remains unaffected. However, as $p_{t}$ approaches 1, the modulating factor reduces the loss, thereby down-weighting well-classified examples and focusing the model’s learning on more challenging cases. When *γ* = 0, focal loss behaves like standard cross-entropy loss. As *γ* increases, the effect of the modulating factor becomes stronger, emphasizing difficult-to-classify samples. Based on prior research [49] and empirical validation experiments, we set *γ* = 2, as this value provided an optimal balance between class imbalance mitigation and model convergence stability.

#### Intervertebral disc label module

##### Disc localization network

A V-Net-based architecture was employed to localize and label intervertebral discs. The final convolutional layer was modified by replacing the number of output channels with the total number of target discs, enabling each channel to represent a specific disc. The channel index corresponded to the anatomical label, and each channel output a heatmap indicating the probability distribution of the disc center.

##### Postprocessing stage

1. For each channel, local maxima with probability values above a predefined threshold were selected as candidate disc centers.
2. A pre-established spatial template of disc positions was then used to constrain and match these candidate points. Specifically, the key reference disc (L5/S1) was first identified, and a sequence-matching algorithm was applied to select the ordered set of candidate centers that minimized the positional error relative to the template.
3. Finally, spatial relationships between vertebral levels were used to fuse the disc segmentation and labeling results, producing final disc-level annotations across all lumbar segments.

This expanded explanation provides full transparency regarding how the labeling module integrates the network output and anatomical constraints to achieve robust, anatomically consistent disc identification (Fig. S5). The formula is expressed as Eq. (3):

$$\begin{array}{c} \text{Matching error}=\sum_{1<i,j<d}\left|D\left(\mathrm{ci},\;\mathrm{cj}\right)-T\left(i,\;j\right)\right| \end{array}$$ (3)

(*d* denotes the number of intervertebral discs. *D*(*ci*, *cj*) represent the spatial coordinates of the candidate points *ci* and *cj* associated with intervertebral discs *i* and *j*, respectively. *T*(*i*, *j*) denotes the empirical distance between intervertebral disc *i* and intervertebral disc *j*.)

The image dataset used for this purpose was collected from the UI Healthcare superconducting MR equipment, comprising 724 training data and 264 validation data from data B for model training, which was conducted over 109 epochs. The final model underwent additional testing on 109 images. The gold standard used for testing was annotated by 2 experts. During the training process, the evaluation utilized mean squared error (MSE) and sensitivity. The MSE formula is expressed as Eq. (4):

$$L\left(Y,\;f\left(x\right)\right)=\frac{1}{N}\sum_{i=1}^{N}{\left(Y_{i}-f\left(x_{i}\right)\right)}^{2}$$ (4)

$Y_{i}$ represents the heat value of each point, and $f\left(x_{i}\right)$ represents the predicted heat value of the corresponding point by the network. *N* denotes the number of pixels. Traditional point localization regression, such as direct coordinate value prediction (*x*, *y*), encounters a fundamental challenge: coordinate regression involves a highly nonlinear mapping, requiring the model to learn a complex transformation from image pixels to absolute numerical values—a task that is inherently difficult. Heatmap-based regression reformulates this problem into one that is semantically more interpretable and easier to learn. Rather than predicting exact coordinates, the approach estimates the likelihood of a point occurring at each pixel location, effectively modeling spatial uncertainty in a probabilistic manner. Moreover, the heatmap was used as the output of the point localization network and generated a Gaussian function value distribution centered around each key point on the heatmap, with a maximum value of 256 (which is beneficial for the network to output more discriminative outputs).

#### Scanning parameter calculation module

The scanning parameter calculation module computes the key points of the target intervertebral discs based on segmentation results. It fits the optimal display sagittal and coronal images, facilitating user observation of the intervertebral disc structure and adjustment of subsequent scanning parameters. Subsequently, on the optimal display coronal and sagittal images, the module employs the least squares method to perform directional fitting for the relevant key points, obtaining the sagittal scan direction and coronal scan direction, and situating the scan center in the desired location.

The algorithm utilizes principal component analysis to calculate the 3D principal direction of each intervertebral disc segmentation mask. It sets the principal direction corresponding to the *Z*-axis (head–foot direction) as the normal vector for each intervertebral disc’s transverse scan slice group and places the scan center at the posterior edge point of each intervertebral disc. The algorithm identifies the most anterior point based on the intervertebral disc segmentation results and, in accordance with the protocol-specified distance and coronal scan direction, determines the location and direction of the saturation band. It calculates the results for each vertebra based on the position, orientation, and label information of adjacent intervertebral discs.

In lumbar spine MRI scanning, the saturation band plays a crucial role in optimizing image quality and diagnostic accuracy [50–52]. The clinical applications of saturation-band placement include the following: (a) Reduction of imaging artifacts: Saturation bands effectively mitigate motion artifacts caused by abdominal respiration and vascular pulsation, leading to improved image clarity and reducing diagnostic ambiguities. (b) Enhancement of lesion visualization: By minimizing motion artifacts, saturation bands improve the detection and visualization of intervertebral disc herniations, vertebral lesions, and intraspinal space-occupying abnormalities, ensuring better diagnostic precision. (c) Optimization of fat suppression: In specific MRI sequences such as short-TI inversion recovery, saturation bands can be combined with fat suppression techniques to enhance lesion contrast and improve detection rates [50–52].

During sagittal lumbar spine imaging, it is essential to position the saturation band sufficiently away from the region of interest to prevent unintentional signal suppression, which could obscure important anatomical details. The model automatically adjusts the positioning and width of the saturation band to balance signal preservation with optimal suppression effects. This adjustment process minimizes signal loss while maintaining effective nulling, ensuring high diagnostic quality and consistent image acquisition across different scanning conditions. The AI-driven placement of the saturation band reduces interoperator variability, streamlining the imaging workflow in both fully automated and AI-assisted MRI acquisitions.

Additionally, the quality factor is an algorithmic measure of reliability, which combines spacing information for all intervertebral discs according to specific calculation rules. It serves as an indicator of the algorithm’s overall reliability [53]. In the LVP system, the quality factor is inversely correlated with model uncertainty—lower uncertainty results in a higher quality factor, indicating greater confidence in the AI-generated scanning parameters.

### Image manual measurements and external evaluation

All MRI data collected from multiple centers were imported into RadiAnt DICOM Viewer (version 2021.1.1, 64-bit, Medixant, Poznan, Poland), which was used to perform manual measurements of imaging parameters. Image measurements were conducted by 2 radiology residents, each with 1 and 1.5 years of experience in MRI, respectively. The measured parameters included offset angle, lateral foramen vertical diameter, disc vertical diameter (actual diameter), psoas muscle cross-sectional area, facet joint angle, degree of disc protrusion/MSU classification, disc herniation ratio, lateral recess height, and lateral recess angle. To ensure measurement proficiency, both residents completed 30 MRI training sessions before the formal assessments. A senior radiologist with over 10 years of experience in spinal MRI interpretation and research subsequently reviewed and validated all measurements. In cases of discrepancies, a 3-expert consensus process was implemented to finalize the measurements. All radiologists were blinded to the automated measurements performed by the LVP system to prevent bias.

To summarize the capabilities of the LVP system, Tables S1 and S2 provide a comprehensive overview of all measurements and assessments produced by the model at each lumbar level. The tables also include the specific measurement techniques, such as cross-sectional area calculations, linear distance measurements, and angular assessments, as previously reported in the literature [54–62].

Positional accuracy assessment: The offset angle at each intervertebral disc level was measured on sagittal localizer images, defined as the angle between the standard positioning line and the actual positioning line [54]. Intervertebral disc height was measured on T1-weighted sagittal images, defined as the vertical distance between the superior and inferior edges of the disc at its midpoint [55]. Foraminal height was assessed on T2-weighted sagittal images, representing the vertical distance between the superior and inferior borders of the foramen [55]. These parameters were used to evaluate MRI scan positioning precision, where smaller offset angles and reduced foraminal/disc height variations indicated closer alignment with standardized anatomical positioning and higher accuracy.

Muscle and joint evaluations: The psoas muscle cross-sectional area at the mid-vertebral level [56] and the facet joint angle at the mid-disc level were measured on axial T2-weighted images. The facet joint angle was defined as the angle between the midline and the left and right facet joints [57]. Left–right differences in these parameters were analyzed to assess overall positioning performance, with smaller asymmetries indicating greater positioning precision.

Spinal canal and disc herniation analysis: On axial T2-weighted images, the MSU classification of disc herniation, disc herniation ratio, spinal canal diameter, lateral recess depth, and lateral recess angle were measured. The MSU classification system categorizes disc herniations based on size (grades 1–2–3) and location (zones A–B–C), resulting in 10 classification types [58,59]. The disc herniation ratio was calculated as the maximum cross-sectional area of the herniated disc divided by the spinal canal area at the same level [60].

Spinal canal diameter was measured as the distance from the midpoint of the posterior edge of the disc to the midpoint of the spinous process at the mid-disc level [61,62]. A diameter <10 mm indicated spinal canal stenosis, and the number of patients diagnosed with this condition in each group was recorded.

Lateral recess depth was defined as the distance between the medial edge of the superior articular process and the posterior edge of the disc [62], with a depth <3 mm indicating lateral recess stenosis.

Lateral recess angle was defined as the angle between 2 lines parallel to the superior and inferior borders of the lateral recess [62], where angles >30° suggested lateral recess stenosis.

The MSU classification and disc herniation ratio were used to evaluate the prevalence and severity of lumbar disc herniation across study groups. Higher diagnostic rates of spinal canal stenosis, lateral recess stenosis, and disc herniation indicated superior positioning accuracy and higher reliability of AI-assisted MRI analysis.

To evaluate the performance of our system, we adopted the detection rate as a key metric. We assumed that the underlying disease prevalence among Chinese patients across different cohorts was consistent, providing a reasonable basis for comparative assessment. Imaging results obtained via different acquisition methods were independently reviewed and evaluated by the same 2 radiologists to ensure consistency in interpretation. In this context, the detection rate—defined as the proportion of positive findings across patient groups—was used as a surrogate for disease prevalence. A higher detection rate was interpreted as an indicator of superior detection performance.

### Data analysis and statistics

The Kolmogorov–Smirnov test was used to analyze whether the data met the normal distribution. If the data did not meet the normal distribution, the nonparametric test was used for analysis. Continuous variables are described as mean ± standard error of the mean. Differences between 2 groups were compared using a 2-sample Student *t* test or the Mann–Whitney *U* test. One-way analysis of variance (ANOVA) and the Kruskal–Wallis test were performed for multiple group comparisons. To control for multiple comparisons, the Bonferroni test was used following one-way ANOVA, and Dunn’s Test was conducted as a post hoc analysis after the Kruskal–Wallis test. The *F* test and *t* test were used to evaluate the difference between the 2 observations. The Bland–Altman method was utilized to assess the consistency between different measurement methods, and this approach was similarly applied to evaluate the consistency of measurements obtained by 2 radiologists. For all statistical analyses, a *P* value <0.05 was considered statistically significant, and analyses were performed using GraphPad PRISM 10.6.

## Data Availability

Original data will be provided by the corresponding authors upon reasonable request.
