# Supplementary material for: Clinical Application of Deep Learning for Spine MRI Interpretation: A Multicenter Evaluation of Artificial-Intelligence-Assisted versus Manual Reading on Diagnostic Agreement with the Reference Standard
Source: Research (Wash D C). 2026 Feb 19;9:1145. doi: 10.34133/research.1145 (PMC12917116; doi:10.34133/research.1145)
Supplement: Supplementary 1 — Tables S1 to S13 Figs. S1 to S5 Movie S1 [file research.1145.f1.zip › SUPPLEMENTARY MATERIALS.docx]

SUPPLEMENTARY MATERIALS

Supplementary Table 1-13

Supplementary Table 1. Manual measurements generated by the LVP system for lumbar spine assessment.

This table specifically describes the measurement indicators, the description of the measurement indicators and the measurement segments of different measurement indicators.

| Measurement metrics | Description | Assessed Levels |
| --- | --- | --- |
| Disc herniation ratio | The ratio of the cross-sectional area of the herniated disc to the cross-sectional area of the spinal canal at the same level, measured on the axial image showing the maximum cross-sectional area of the disc herniation | L1/2 - L5/S1 |
| Psoas area | The cross-sectional area of the psoas major muscle | L1 - L5 |
| Facet joint angle | The angle formed by the intersection of the line connecting the anteromedial and posterolateral ends of the zygapophyseal joint and the mid-sagittal line passing through the spinous process | L1/2 - L5/S1 |
| Intervertebral disc  vertical diameter | The vertical distance between the superior and inferior edges of the intervertebral disc measured at the center of the disc | L1/2 - L5/S1 |
| Foraminal  vertical diameter | The distance between the pedicles measured at the sagittal level of the intervertebral foramen | L1/2 - L5/S1 |
| Anteroposterior  diameter of spinal  canal | The distance between the posterior edge of the intervertebral disc and the ligamentum flavum measured at the axial level of the intervertebral disc | L1/2 - L5/S1 |
| Lateral recess height | The distance between the superior articular facet and the top part of the pedicle | L1/2 - L5/S1 |
| Lateral recess angle | The angle between the bottom and the roof of the triangular space | L1/2 - L5/S1 |
| Offset angle | The angle formed between the positioning line and the standard reference line on the sagittal localizer image | L1/2 - L5/S1 |
| Disc Protrusion  Classification | MSU classification | L1/2 - L5/S1 |

Supplementary Table 2. The measured values for different imaging parameters.

This table shows the specific measurements of DL, HM and FM for different indicators. DL: deep learning group, HM: human-machine group, FM: fully manual group.

|  | DL | HM | FM | DL | HM | FM |
| --- | --- | --- | --- | --- | --- | --- |
|  | Offset angle (°) | | | Psoas area (cm2) | | |
| L1/2 | 0.85±0.92 | 1.13±0.74 | 1.79±1.58 | 0.32±0.61 | 0.34±0.81 | 0.57±5.48 |
| L2/3 | 0.90±0.89 | 1.13±0.82 | 1.69±1.46 | 0.58±0.57 | 0.55±0.44 | 0.77±2.61 |
| L3/4 | 0.85±0.97 | 1.07±0.78 | 1.63±1.39 | 0.81±0.72 | 0.65±0.58 | 0.86±0.81 |
| L4/5 | 1.11±1.16 | 1.09±0.76 | 1.78±1.51 | 0.91±0.80 | 1.49±7.58 | 1.14±3.99 |
| L5/1 | 1.91±1.88 | 1.77±1.25 | 3.06±2.74 | 1.02±0.92 | 0.91±0.81 | 1.22±3.24 |
|  | Facet joint angle (°) | | | Disc height (cm) | | |
| L1/2 | 5.72±4.71 | 6.03±4.50 | 7.42±6.07 | 0.84±0.15 | 0.81±0.18 | 0.94±0.38 |
| L2/3 | 5.69±4.81 | 5.82±4.66 | 7.53±5.90 | 0.97±0.18 | 0.94±0.21 | 1.04±0.29 |
| L3/4 | 5.66±4.54 | 5.43±4.20 | 7.58±6.43 | 1.08±0.20 | 1.08±0.19 | 1.15±0.21 |
| L4/5 | 5.78±4.20 | 9.04±29.88 | 7.59±9.31 | 1.10±0.22 | 1.09±0.23 | 1.15±0.25 |
| L5/1 | 5.75±4.48 | 5.96±4.56 | 7.49±6.25 | 1.02±0.24 | 1.07±0.65 | 1.08±0.27 |
|  | Left lateral foramen vertical diameter (cm) | | | Right lateral foramen vertical diameter (cm) | | |
| L1/2 | 1.77±0.21 | 1.78±0.24 | 1.74±0.25 | 1.78±0.21 | 1.80±0.21 | 1.75±0.24 |
| L2/3 | 1.86±0.25 | 1.88±0.30 | 1.83±0.31 | 1.86±0.28 | 1.90±0.28 | 1.85±0.30 |
| L3/4 | 1.80±0.29 | 1.87±0.27 | 1.82±0.30 | 1.80±0.29 | 1.87±0.29 | 1.82±0.30 |
| L4/5 | 1.66±0.28 | 1.71±0.26 | 1.64±0.30 | 1.65±0.26 | 1.70±0.28 | 1.65±0.41 |
| L5/1 | 1.41±0.23 | 1.40±0.23 | 1.38±0.24 | 1.41±0.23 | 1.44±0.22 | 1.40±0.23 |
|  | Spinal canal diameter (cm) | | | Lumbar disc herniation ratio (%) | | |
| L1/2 | 2.06±0.21 | 1.99±0.21 | 1.97±0.26 | 0.01±0.05 | 0.02±0.07 | 0.02±0.07 |
| L2/3 | 2.00±0.24 | 1.90±0.23 | 1.96±0.28 | 0.05±0.11 | 0.06±0.13 | 0.05±0.12 |
| L3/4 | 1.89±0.28 | 1.79±0.29 | 1.87±0.32 | 0.13±0.16 | 0.14±0.17 | 0.12±0.17 |
| L4/5 | 1.63±0.29 | 1.55±0.28 | 1.63±0.33 | 0.22±0.17 | 0.23±0.19 | 0.24±0.20 |
| L5/1 | 1.53±0.29 | 1.43±0.26 | 1.48±0.30 | 0.10±0.15 | 0.12±0.14 | 0.16±1.03 |
|  | Left lateral recess height (mm) | | | Right lateral recess height (mm) | | |
| L1/2 | 4.77±1.39 | 4.80±1.46 | 5.19±1.49 | 5.16±1.39 | 5.02±1.54 | 5.57±2.30 |
| L2/3 | 3.62±1.41 | 3.67±1.54 | 4.21±1.58 | 3.91±1.53 | 3.64±1.52 | 4.41±1.61 |
| L3/4 | 2.61±1.24 | 2.55±1.42 | 3.19±1.54 | 2.66±1.29 | 2.52±1.42 | 3.30 ±2.55 |
| L4/5 | 1.86±1.10 | 1.67±1.22 | 2.09±1.36 | 1.89±1.17 | 1.71±1.12 | 2.10±1.38 |
| L5/1 | 2.96±1.42 | 2.39±1.52 | 2.97±1.52 | 3.01±1.45 | 2.62±1.96 | 3.02±1.54 |
|  | Left lateral recess angle (°) | | | Left lateral recess angle (°) | | |
| L1/2 | 34.86±4.91 | 34.81±6.10 | 35.87±5.21 | 35.69±4.35 | 33.52±5.01 | 35.86±12.82 |
| L2/3 | 31.10±8.15 | 30.55±9.36 | 33.56±7.15 | 33.58±7.19 | 31.19±8.77 | 34.07±7.10 |
| L3/4 | 26.54±8.92 | 25.88±9.91 | 29.58±9.62 | 28.46±9.82 | 26.44±10.12 | 29.78±10.09 |
| L4/5 | 21.82±9.00 | 20.83±9.56 | 23.85±10.35 | 22.80±9.10 | 21.89±9.25 | 24.27±10.80 |
| L5/1 | 31.01±19.91 | 25.87±9.70 | 29.97±10.46 | 29.96±9.25 | 26.27±9.97 | 30.10±10.63 |

Supplementary Table 3. Experiment result record table of the best displaying sagittal image

Experiment result record table of the best displaying sagittal image, which records the number of target discs in the best displaying sagittal image selected manually (abbreviated as NM) and the number of target discs in the best displaying sagittal image output by the algorithm (abbreviated as NA).

| ID | NM | NA | ID | NM | NA | ID | NM | NA | ID | NM | NA | ID | NM | NA |
| --- | --- | --- | --- | --- | --- | --- | --- | --- | --- | --- | --- | --- | --- | --- |
| 1 | 5 | 5 | 21 | 4 | 4 | 41 | 6 | 6 | 61 | 10 | 10 | 81 | 10 | 10 |
| 2 | 8 | 8 | 22 | 5 | 5 | 42 | 7 | 7 | 62 | 10 | 10 | 82 | 6 | 6 |
| 3 | 7 | 7 | 23 | 5 | 5 | 43 | 4 | 4 | 63 | 10 | 10 | 83 | 6 | 6 |
| 4 | 6 | 6 | 24 | 4 | 4 | 44 | 5 | 5 | 64 | 10 | 10 | 84 | 6 | 6 |
| 5 | 9 | 9 | 25 | 5 | 5 | 45 | 7 | 7 | 65 | 9 | 9 | 85 | 8 | 8 |
| 6 | 7 | 7 | 26 | 5 | 5 | 46 | 7 | 7 | 66 | 10 | 10 | 86 | 10 | 10 |
| 7 | 8 | 8 | 27 | 4 | 4 | 47 | 4 | 4 | 67 | 10 | 10 | 87 | 10 | 10 |
| 8 | 4 | 4 | 28 | 6 | 6 | 48 | 4 | 4 | 68 | 9 | 9 | 88 | 8 | 8 |
| 9 | 4 | 4 | 29 | 4 | 4 | 49 | 6 | 6 | 69 | 10 | 10 | 89 | 10 | 10 |
| 10 | 4 | 4 | 30 | 9 | 9 | 50 | 4 | 4 | 70 | 10 | 10 | 90 | 6 | 6 |
| 11 | 4 | 4 | 31 | 4 | 4 | 51 | 7 | 7 | 71 | 10 | 10 | 91 | 7 | 7 |
| 12 | 4 | 4 | 32 | 7 | 7 | 52 | 8 | 8 | 72 | 7 | 7 | 92 | 6 | 6 |
| 13 | 4 | 4 | 33 | 7 | 7 | 53 | 8 | 8 | 73 | 8 | 8 | 93 | 11 | 11 |
| 14 | 4 | 4 | 34 | 7 | 7 | 54 | 7 | 7 | 74 | 8 | 8 | 94 | 10 | 10 |
| 15 | 5 | 5 | 35 | 6 | 6 | 55 | 6 | 6 | 75 | 8 | 8 | 95 | 8 | 8 |
| 16 | 4 | 4 | 36 | 4 | 4 | 56 | 7 | 7 | 76 | 7 | 7 | 96 | 9 | 9 |
| 17 | 5 | 5 | 37 | 4 | 4 | 57 | 6 | 6 | 77 | 10 | 10 | 97 | 9 | 9 |
| 18 | 6 | 6 | 38 | 6 | 6 | 58 | 10 | 10 | 78 | 7 | 7 | 98 | 8 | 8 |
| 19 | 4 | 4 | 39 | 4 | 4 | 59 | 10 | 10 | 79 | 10 | 10 | 99 | 6 | 6 |
| 20 | 5 | 5 | 40 | 4 | 4 | 60 | 10 | 10 | 80 | 10 | 10 | 100 | 8 | 8 |

Supplementary Table 4. Experiment result record table of the best displaying coronal image

Experiment result record table of the best displaying coronal image, which records the number of target discs in the best displaying coronal image selected manually (abbreviated as NM) and the number of target discs in the best displaying coronal image output by the algorithm (abbreviated as NA).

| ID | NM | NA | ID | NM | NA | ID | NM | NA | ID | NM | NA | ID | NM | NA |
| --- | --- | --- | --- | --- | --- | --- | --- | --- | --- | --- | --- | --- | --- | --- |
| 1 | 5 | 5 | 21 | 4 | 4 | 41 | 6 | 6 | 61 | 6 | 6 | 81 | 6 | 6 |
| 2 | 6 | 6 | 22 | 5 | 5 | 42 | 7 | 7 | 62 | 6 | 6 | 82 | 6 | 6 |
| 3 | 6 | 6 | 23 | 5 | 5 | 43 | 4 | 4 | 63 | 6 | 6 | 83 | 6 | 6 |
| 4 | 6 | 6 | 24 | 4 | 4 | 44 | 5 | 5 | 64 | 6 | 6 | 84 | 6 | 6 |
| 5 | 6 | 6 | 25 | 5 | 5 | 45 | 6 | 6 | 65 | 6 | 6 | 85 | 6 | 6 |
| 6 | 6 | 6 | 26 | 5 | 5 | 46 | 6 | 6 | 66 | 6 | 6 | 86 | 6 | 6 |
| 7 | 6 | 6 | 27 | 4 | 4 | 47 | 4 | 4 | 67 | 6 | 6 | 87 | 6 | 6 |
| 8 | 4 | 4 | 28 | 6 | 6 | 48 | 4 | 4 | 68 | 6 | 6 | 88 | 6 | 6 |
| 9 | 4 | 4 | 29 | 4 | 4 | 49 | 6 | 6 | 69 | 6 | 6 | 89 | 6 | 6 |
| 10 | 4 | 4 | 30 | 6 | 6 | 50 | 4 | 4 | 70 | 6 | 6 | 90 | 6 | 6 |
| 11 | 4 | 4 | 31 | 4 | 4 | 51 | 6 | 6 | 71 | 6 | 6 | 91 | 6 | 6 |
| 12 | 4 | 4 | 32 | 6 | 6 | 52 | 6 | 6 | 72 | 6 | 6 | 92 | 6 | 6 |
| 13 | 4 | 4 | 33 | 6 | 6 | 53 | 6 | 6 | 73 | 6 | 6 | 93 | 6 | 6 |
| 14 | 4 | 4 | 34 | 6 | 6 | 54 | 6 | 6 | 74 | 6 | 6 | 94 | 6 | 6 |
| 15 | 5 | 5 | 35 | 6 | 6 | 55 | 6 | 6 | 75 | 6 | 6 | 95 | 6 | 6 |
| 16 | 4 | 4 | 36 | 4 | 4 | 56 | 6 | 6 | 76 | 6 | 6 | 96 | 6 | 6 |
| 17 | 5 | 5 | 37 | 4 | 4 | 57 | 6 | 6 | 77 | 6 | 6 | 97 | 6 | 6 |
| 18 | 6 | 6 | 38 | 6 | 6 | 58 | 6 | 6 | 78 | 6 | 6 | 98 | 6 | 6 |
| 19 | 4 | 4 | 39 | 4 | 4 | 59 | 6 | 6 | 79 | 6 | 6 | 99 | 6 | 6 |
| 20 | 5 | 5 | 40 | 7 | 7 | 60 | 6 | 6 | 80 | 6 | 6 | 100 | 6 | 6 |

Supplementary Table 5. Error record table of sagittal scanning center and direction

Error record table of sagittal scanning center and direction, which records the angle error of direction annotated between two testers (abbreviated as dirET), the angle error between the direction of algorithm result and gold standard (abbreviated as dirEA), and the distance error between the center point of algorithm result and gold standard (abbreviated as cenEA).

| ID | dirET  (°) | dirEA (°) | cenEA (mm) | ID | dirET  (°) | dirEA (°) | cenEA (mm) | ID | dirET  (°) | dirEA (°) | cenEA (mm) |
| --- | --- | --- | --- | --- | --- | --- | --- | --- | --- | --- | --- |
| 1 | 1.32 | 1.23 | 2.67 | 35 | 0.93 | 0.21 | 3.84 | 69 | 3.61 | 0.21 | 1.19 |
| 2 | 0.19 | 0.09 | 2.93 | 36 | 1.02 | 0.28 | 2.70 | 70 | 3.70 | 0.60 | 2.62 |
| 3 | 0.00 | 0.14 | 1.70 | 37 | 1.43 | 0.05 | 2.95 | 71 | 0.31 | 0.00 | 2.22 |
| 4 | 0.75 | 0.61 | 3.09 | 38 | 0.57 | 0.88 | 1.84 | 72 | 1.10 | 1.20 | 1.41 |
| 5 | 0.56 | 0.60 | 4.78 | 39 | 1.40 | 0.81 | 4.40 | 73 | 0.04 | 0.27 | 2.21 |
| 6 | 1.07 | 0.23 | 2.94 | 40 | 0.05 | 0.09 | 3.40 | 74 | 3.07 | 1.81 | 1.03 |
| 7 | 0.43 | 0.05 | 2.68 | 41 | 0.23 | 0.08 | 4.66 | 75 | 5.95 | 2.15 | 1.69 |
| 8 | 3.84 | 1.34 | 2.25 | 42 | 0.48 | 0.64 | 1.82 | 76 | 0.20 | 1.47 | 2.96 |
| 9 | 0.07 | 2.80 | 4.15 | 43 | 1.45 | 5.09 | 7.86 | 77 | 1.89 | 1.27 | 1.28 |
| 10 | 2.74 | 0.87 | 3.91 | 44 | 1.81 | 0.77 | 3.15 | 78 | 1.09 | 0.78 | 0.85 |
| 11 | 2.09 | 1.49 | 1.68 | 45 | 0.07 | 0.52 | 3.78 | 79 | 0.64 | 0.24 | 1.65 |
| 12 | 0.33 | 0.37 | 1.42 | 46 | 0.63 | 0.94 | 2.55 | 80 | 1.61 | 1.02 | 4.06 |
| 13 | 0.44 | 0.48 | 3.79 | 47 | 2.66 | 0.31 | 2.52 | 81 | 1.17 | 0.57 | 2.60 |
| 14 | 1.55 | 0.87 | 3.25 | 48 | 0.84 | 1.12 | 1.32 | 82 | 1.01 | 2.35 | 1.71 |
| 15 | 0.45 | 1.49 | 3.30 | 49 | 0.16 | 0.27 | 2.42 | 83 | 0.29 | 0.66 | 1.65 |
| 16 | 1.98 | 0.55 | 3.64 | 50 | 1.33 | 0.76 | 3.25 | 84 | 0.51 | 1.72 | 2.39 |
| 17 | 2.39 | 1.64 | 1.97 | 51 | 0.72 | 0.08 | 2.26 | 85 | 0.13 | 0.42 | 1.05 |
| 18 | 1.47 | 0.96 | 4.18 | 52 | 1.86 | 1.55 | 1.01 | 86 | 2.09 | 0.75 | 1.81 |
| 19 | 2.69 | 0.72 | 3.44 | 53 | 1.07 | 0.36 | 1.88 | 87 | 0.58 | 1.06 | 1.93 |
| 20 | 0.07 | 1.12 | 4.07 | 54 | 1.24 | 0.09 | 2.50 | 88 | 0.27 | 0.57 | 1.85 |
| 21 | 1.48 | 0.56 | 3.78 | 55 | 0.18 | 1.26 | 2.17 | 89 | 0.59 | 2.02 | 2.13 |
| 22 | 0.30 | 1.22 | 2.04 | 56 | 1.49 | 2.13 | 3.19 | 90 | 0.81 | 0.12 | 1.02 |
| 23 | 0.43 | 2.12 | 3.02 | 57 | 2.04 | 0.21 | 2.37 | 91 | 1.85 | 0.00 | 2.64 |
| 24 | 2.23 | 0.26 | 3.20 | 58 | 0.69 | 2.16 | 3.02 | 92 | 0.26 | 0.26 | 1.37 |
| 25 | 1.25 | 0.05 | 2.34 | 59 | 0.73 | 1.21 | 1.95 | 93 | 0.19 | 0.27 | 1.62 |
| 26 | 1.34 | 0.08 | 1.11 | 60 | 0.09 | 1.47 | 3.41 | 94 | 0.85 | 0.60 | 2.99 |
| 27 | 0.04 | 0.64 | 3.05 | 61 | 1.18 | 0.86 | 2.38 | 95 | 0.59 | 1.97 | 2.76 |
| 28 | 0.21 | 1.24 | 5.02 | 62 | 0.90 | 1.43 | 2.65 | 96 | 0.48 | 0.86 | 1.51 |
| 29 | 0.46 | 0.05 | 4.85 | 63 | 2.43 | 0.16 | 0.99 | 97 | 1.42 | 0.57 | 0.62 |
| 30 | 0.00 | 0.34 | 3.76 | 64 | 0.27 | 0.65 | 2.10 | 98 | 0.09 | 1.22 | 2.18 |
| 31 | 0.38 | 0.64 | 4.63 | 65 | 0.84 | 1.22 | 2.90 | 99 | 0.65 | 0.86 | 1.89 |
| 32 | 2.47 | 1.15 | 4.02 | 66 | 0.97 | 0.36 | 1.27 | 100 | 0.86 | 1.24 | 1.98 |
| 33 | 1.21 | 1.24 | 2.44 | 67 | 0.64 | 0.34 | 2.89 | mean | 1.09 | 0.86 | 2.64 |
| 34 | 0.28 | 0.51 | 2.97 | 68 | 2.03 | 0.48 | 3.18 | - | - | - | - |

Supplementary Table 6. Error record table of coronal scanning center and direction

Error record table of coronal scanning center and direction, which records the angle error of direction annotated between two testers (abbreviated as dirET), the angle error between the direction of algorithm result and gold standard (abbreviated as dirEA), and the distance error between the center point of algorithm result and gold standard (abbreviated as cenEA).

| ID | dirET  (°) | dirEA (°) | cenEA (mm) | ID | dirET  (°) | dirEA (°) | cenEA (mm) | ID | dirET  (°) | dirEA (°) | cenEA (mm) |
| --- | --- | --- | --- | --- | --- | --- | --- | --- | --- | --- | --- |
| 1 | 3.27 | 2.66 | 4.69 | 35 | 2.64 | 1.43 | 4.78 | 69 | 2.71 | 1.07 | 2.05 |
| 2 | 0.70 | 0.93 | 4.09 | 36 | 2.64 | 1.18 | 2.63 | 70 | 0.17 | 1.85 | 2.98 |
| 3 | 0.86 | 1.27 | 2.24 | 37 | 0.49 | 0.97 | 2.49 | 71 | 1.93 | 0.46 | 1.78 |
| 4 | 1.05 | 0.97 | 5.74 | 38 | 4.20 | 0.88 | 2.46 | 72 | 0.03 | 2.04 | 2.93 |
| 5 | 2.47 | 1.40 | 3.91 | 39 | 3.05 | 0.07 | 4.30 | 73 | 0.26 | 2.73 | 2.28 |
| 6 | 3.02 | 1.32 | 3.47 | 40 | 1.03 | 1.77 | 3.01 | 74 | 0.62 | 3.15 | 1.57 |
| 7 | 2.61 | 1.58 | 3.70 | 41 | 0.86 | 0.74 | 5.58 | 75 | 5.73 | 1.00 | 1.61 |
| 8 | 2.76 | 1.15 | 2.07 | 42 | 1.19 | 1.90 | 1.74 | 76 | 1.66 | 0.37 | 1.61 |
| 9 | 3.92 | 0.75 | 3.14 | 43 | 0.62 | 3.31 | 6.84 | 77 | 1.88 | 0.42 | 1.51 |
| 10 | 1.14 | 1.66 | 2.67 | 44 | 1.56 | 1.19 | 4.10 | 78 | 0.80 | 0.34 | 1.09 |
| 11 | 2.46 | 0.54 | 1.96 | 45 | 2.69 | 0.05 | 4.02 | 79 | 2.62 | 0.15 | 1.85 |
| 12 | 2.85 | 2.87 | 2.63 | 46 | 1.91 | 0.50 | 3.58 | 80 | 6.01 | 0.16 | 2.91 |
| 13 | 0.36 | 1.81 | 4.07 | 47 | 2.33 | 2.36 | 2.64 | 81 | 4.19 | 1.12 | 3.27 |
| 14 | 4.02 | 0.76 | 2.93 | 48 | 1.13 | 1.28 | 1.22 | 82 | 0.11 | 0.21 | 2.53 |
| 15 | 2.64 | 6.40 | 5.40 | 49 | 0.84 | 1.11 | 2.10 | 83 | 1.01 | 1.94 | 2.95 |
| 16 | 3.29 | 1.69 | 4.78 | 50 | 2.42 | 0.29 | 3.74 | 84 | 3.54 | 0.13 | 2.60 |
| 17 | 2.51 | 0.88 | 1.89 | 51 | 1.65 | 0.00 | 2.14 | 85 | 1.43 | 0.42 | 1.21 |
| 18 | 0.22 | 0.87 | 5.74 | 52 | 1.42 | 3.60 | 1.30 | 86 | 0.26 | 0.81 | 1.30 |
| 19 | 4.76 | 0.63 | 3.55 | 53 | 0.11 | 1.58 | 1.75 | 87 | 3.32 | 2.19 | 1.33 |
| 20 | 4.01 | 0.64 | 3.30 | 54 | 1.12 | 1.10 | 1.56 | 88 | 2.24 | 0.00 | 2.86 |
| 21 | 3.35 | 0.56 | 3.70 | 55 | 2.10 | 1.49 | 1.59 | 89 | 1.19 | 4.96 | 3.27 |
| 22 | 1.37 | 1.39 | 5.01 | 56 | 1.84 | 1.32 | 3.82 | 90 | 1.25 | 0.02 | 1.27 |
| 23 | 0.87 | 2.17 | 3.62 | 57 | 1.13 | 1.66 | 3.59 | 91 | 2.02 | 2.20 | 4.58 |
| 24 | 1.51 | 0.79 | 4.20 | 58 | 3.08 | 3.97 | 1.48 | 92 | 0.49 | 1.29 | 2.19 |
| 25 | 0.02 | 0.87 | 3.29 | 59 | 0.51 | 1.66 | 0.60 | 93 | 2.45 | 2.51 | 3.27 |
| 26 | 0.92 | 0.10 | 1.06 | 60 | 3.32 | 4.45 | 2.21 | 94 | 4.47 | 3.10 | 2.22 |
| 27 | 0.84 | 1.84 | 3.24 | 61 | 0.49 | 0.71 | 3.42 | 95 | 4.07 | 3.01 | 2.93 |
| 28 | 0.24 | 0.37 | 2.45 | 62 | 1.54 | 0.93 | 1.52 | 96 | 2.56 | 2.65 | 1.19 |
| 29 | 2.74 | 1.24 | 5.34 | 63 | 0.43 | 0.14 | 2.68 | 97 | 1.64 | 2.84 | 3.82 |
| 30 | 1.49 | 0.04 | 3.57 | 64 | 0.80 | 0.00 | 0.67 | 98 | 2.42 | 1.62 | 2.28 |
| 31 | 1.94 | 0.72 | 3.71 | 65 | 0.09 | 1.64 | 1.69 | 99 | 0.30 | 0.81 | 1.90 |
| 32 | 1.58 | 0.27 | 4.51 | 66 | 1.08 | 1.53 | 4.79 | 100 | 0.37 | 0.15 | 1.95 |
| 33 | 3.21 | 0.03 | 3.88 | 67 | 0.42 | 0.08 | 0.57 | mean | 1.86 | 1.33 | 2.88 |
| 34 | 0.50 | 0.90 | 2.70 | 68 | 1.64 | 0.43 | 2.23 | - | - | - | - |

Supplementary Table 7. Error record table of transverse scanning center and direction

Error record table of transverse scanning center and direction, which records the angle error of direction annotated between two testers (abbreviated as dirET), the angle error between the direction of algorithm result and gold standard (abbreviated as dirEA), and the distance error between the center point of algorithm result and gold standard (abbreviated as cenEA).

| ID | dirET  (°) | dirEA (°) | cenEA (mm) | ID | dirET  (°) | dirEA (°) | cenEA (mm) | ID | dirET  (°) | dirEA (°) | cenEA (mm) |
| --- | --- | --- | --- | --- | --- | --- | --- | --- | --- | --- | --- |
| 1 | 1.85 | 0.87 | 2.91 | 35 | 0.91 | 1.83 | 3.54 | 69 | 3.12 | 2.44 | 2.35 |
| 2 | 1.65 | 0.90 | 5.82 | 36 | 1.84 | 1.70 | 2.96 | 70 | 2.37 | 2.70 | 1.99 |
| 3 | 0.92 | 1.53 | 2.10 | 37 | 1.48 | 0.72 | 2.85 | 71 | 2.50 | 2.38 | 2.29 |
| 4 | 1.85 | 1.68 | 4.34 | 38 | 1.54 | 1.92 | 2.44 | 72 | 3.26 | 2.63 | 2.04 |
| 5 | 1.82 | 1.12 | 2.39 | 39 | 1.74 | 1.19 | 3.58 | 73 | 2.07 | 3.02 | 2.06 |
| 6 | 1.62 | 1.79 | 2.76 | 40 | 1.57 | 1.59 | 1.86 | 74 | 3.28 | 2.62 | 1.91 |
| 7 | 1.42 | 0.70 | 2.92 | 41 | 2.35 | 2.00 | 2.16 | 75 | 2.27 | 2.49 | 2.53 |
| 8 | 2.01 | 2.05 | 2.82 | 42 | 1.52 | 1.53 | 2.35 | 76 | 2.06 | 2.75 | 2.35 |
| 9 | 1.43 | 1.09 | 3.20 | 43 | 2.61 | 1.50 | 3.94 | 77 | 1.98 | 2.50 | 2.71 |
| 10 | 1.28 | 1.32 | 3.38 | 44 | 2.44 | 1.17 | 3.00 | 78 | 2.94 | 1.62 | 2.43 |
| 11 | 1.03 | 1.65 | 2.06 | 45 | 1.78 | 1.76 | 2.60 | 79 | 1.52 | 1.84 | 3.76 |
| 12 | 2.76 | 2.03 | 2.65 | 46 | 1.77 | 1.26 | 2.77 | 80 | 1.88 | 2.54 | 2.43 |
| 13 | 1.33 | 1.08 | 3.52 | 47 | 1.48 | 1.89 | 2.10 | 81 | 2.01 | 2.61 | 1.88 |
| 14 | 1.10 | 2.16 | 3.19 | 48 | 1.38 | 1.28 | 1.71 | 82 | 3.25 | 2.85 | 2.78 |
| 15 | 1.83 | 1.62 | 3.45 | 49 | 2.60 | 1.67 | 3.25 | 83 | 3.31 | 3.34 | 2.61 |
| 16 | 1.51 | 1.71 | 3.04 | 50 | 2.76 | 1.72 | 3.66 | 84 | 3.18 | 2.58 | 1.41 |
| 17 | 1.37 | 2.33 | 2.86 | 51 | 2.81 | 2.67 | 1.03 | 85 | 2.16 | 3.52 | 1.87 |
| 18 | 1.00 | 1.38 | 4.47 | 52 | 2.78 | 2.19 | 0.90 | 86 | 3.49 | 2.41 | 1.88 |
| 19 | 1.68 | 1.96 | 3.79 | 53 | 1.61 | 1.79 | 1.00 | 87 | 2.04 | 2.19 | 2.26 |
| 20 | 1.62 | 1.74 | 3.48 | 54 | 3.40 | 2.41 | 2.73 | 88 | 2.53 | 2.90 | 1.98 |
| 21 | 1.02 | 1.18 | 3.19 | 55 | 1.80 | 2.77 | 1.45 | 89 | 2.33 | 3.65 | 3.09 |
| 22 | 1.16 | 1.99 | 3.48 | 56 | 5.31 | 2.24 | 1.61 | 90 | 2.46 | 1.82 | 1.61 |
| 23 | 1.77 | 1.17 | 1.85 | 57 | 2.48 | 2.02 | 1.15 | 91 | 2.32 | 2.52 | 1.63 |
| 24 | 1.26 | 2.24 | 3.16 | 58 | 2.26 | 2.28 | 1.95 | 92 | 3.00 | 2.82 | 1.11 |
| 25 | 1.22 | 1.91 | 3.46 | 59 | 2.24 | 1.30 | 1.77 | 93 | 1.66 | 2.33 | 1.34 |
| 26 | 1.53 | 1.00 | 1.99 | 60 | 2.23 | 1.86 | 1.69 | 94 | 2.40 | 3.33 | 1.24 |
| 27 | 1.50 | 1.60 | 3.12 | 61 | 2.25 | 2.52 | 1.58 | 95 | 2.48 | 3.23 | 2.31 |
| 28 | 0.83 | 1.34 | 2.70 | 62 | 3.13 | 2.97 | 2.30 | 96 | 3.10 | 2.99 | 1.39 |
| 29 | 0.77 | 2.85 | 4.83 | 63 | 2.07 | 1.54 | 2.06 | 97 | 2.81 | 2.73 | 1.64 |
| 30 | 1.44 | 1.24 | 2.83 | 64 | 1.91 | 2.22 | 1.40 | 98 | 2.04 | 3.88 | 2.20 |
| 31 | 1.39 | 1.34 | 3.27 | 65 | 2.62 | 2.81 | 1.78 | 99 | 2.64 | 2.26 | 1.30 |
| 32 | 0.98 | 1.26 | 3.66 | 66 | 1.77 | 2.07 | 2.38 | 100 | 2.42 | 3.51 | 1.45 |
| 33 | 1.23 | 0.63 | 2.90 | 67 | 2.42 | 2.16 | 2.25 | mean | 2.04 | 2.03 | 2.50 |
| 34 | 0.99 | 1.26 | 2.86 | 68 | 1.93 | 2.18 | 2.37 | - | - | - | - |

Supplementary Table 8. Scoring record table

Scoring record table of saturated band

| ID | Sc | ID | Sc | ID | Sc | ID | Sc | ID | Sc | ID | Sc | ID | Sc | ID | Sc |
| --- | --- | --- | --- | --- | --- | --- | --- | --- | --- | --- | --- | --- | --- | --- | --- |
| 1 | 3 | 14 | 3 | 27 | 3 | 40 | 3 | 53 | 3 | 66 | 3 | 79 | 3 | 92 | 3 |
| 2 | 3 | 15 | 3 | 28 | 3 | 41 | 3 | 54 | 3 | 67 | 3 | 80 | 3 | 93 | 3 |
| 3 | 3 | 16 | 3 | 29 | 3 | 42 | 3 | 55 | 3 | 68 | 3 | 81 | 3 | 94 | 3 |
| 4 | 3 | 17 | 3 | 30 | 3 | 43 | 3 | 56 | 3 | 69 | 3 | 82 | 3 | 95 | 3 |
| 5 | 3 | 18 | 3 | 31 | 3 | 44 | 3 | 57 | 3 | 70 | 3 | 83 | 3 | 96 | 3 |
| 6 | 3 | 19 | 3 | 32 | 3 | 45 | 3 | 58 | 3 | 71 | 3 | 84 | 3 | 97 | 3 |
| 7 | 3 | 20 | 3 | 33 | 3 | 46 | 3 | 59 | 3 | 72 | 3 | 85 | 3 | 98 | 3 |
| 8 | 3 | 21 | 3 | 34 | 3 | 47 | 3 | 60 | 3 | 73 | 3 | 86 | 3 | 99 | 3 |
| 9 | 3 | 22 | 3 | 35 | 3 | 48 | 3 | 61 | 3 | 74 | 3 | 87 | 3 | 100 | 3 |
| 10 | 3 | 23 | 3 | 36 | 3 | 49 | 3 | 62 | 3 | 75 | 3 | 88 | 3 | Mean | 3 |
| 11 | 3 | 24 | 3 | 37 | 3 | 50 | 3 | 63 | 3 | 76 | 3 | 89 | 3 | - | - |
| 12 | 3 | 25 | 3 | 38 | 3 | 51 | 3 | 64 | 3 | 77 | 3 | 90 | 3 | - | - |
| 13 | 3 | 26 | 3 | 39 | 3 | 52 | 3 | 65 | 3 | 78 | 3 | 91 | 3 | - | - |

Supplementary Table 9. Intervertebral disc label result record table

Intervertebral disc label result record table, which records the actual number of discs (abbreviated as Nact) and the number of disc labels correctly identified by algorithm (abbreviated as Ncorr) in each case.

| ID | Nact | Ncorr | ID | Nact | Ncorr | ID | Nact | Ncorr | ID | Nact | Ncorr | ID | Nact | Ncorr |
| --- | --- | --- | --- | --- | --- | --- | --- | --- | --- | --- | --- | --- | --- | --- |
| 1 | 5 | 5 | 21 | 4 | 4 | 41 | 6 | 6 | 61 | 12 | 12 | 81 | 11 | 11 |
| 2 | 8 | 8 | 22 | 5 | 5 | 42 | 7 | 7 | 62 | 11 | 11 | 82 | 6 | 6 |
| 3 | 7 | 7 | 23 | 5 | 5 | 43 | 4 | 4 | 63 | 12 | 12 | 83 | 6 | 6 |
| 4 | 6 | 6 | 24 | 4 | 4 | 44 | 5 | 5 | 64 | 11 | 11 | 84 | 6 | 6 |
| 5 | 9 | 9 | 25 | 5 | 5 | 45 | 7 | 7 | 65 | 9 | 9 | 85 | 8 | 8 |
| 6 | 7 | 7 | 26 | 5 | 5 | 46 | 6 | 6 | 66 | 12 | 12 | 86 | 12 | 12 |
| 7 | 8 | 8 | 27 | 4 | 4 | 47 | 4 | 4 | 67 | 11 | 11 | 87 | 12 | 12 |
| 8 | 4 | 4 | 28 | 6 | 6 | 48 | 4 | 4 | 68 | 9 | 9 | 88 | 8 | 8 |
| 9 | 4 | 4 | 29 | 4 | 4 | 49 | 6 | 6 | 69 | 12 | 12 | 89 | 11 | 11 |
| 10 | 4 | 4 | 30 | 9 | 9 | 50 | 4 | 4 | 70 | 10 | 10 | 90 | 6 | 6 |
| 11 | 4 | 4 | 31 | 4 | 4 | 51 | 7 | 7 | 71 | 11 | 11 | 91 | 7 | 7 |
| 12 | 4 | 4 | 32 | 7 | 7 | 52 | 7 | 7 | 72 | 7 | 7 | 92 | 6 | 6 |
| 13 | 4 | 4 | 33 | 7 | 7 | 53 | 8 | 8 | 73 | 8 | 8 | 93 | 11 | 11 |
| 14 | 4 | 4 | 34 | 7 | 7 | 54 | 7 | 7 | 74 | 8 | 8 | 94 | 10 | 10 |
| 15 | 6 | 6 | 35 | 6 | 6 | 55 | 6 | 6 | 75 | 8 | 8 | 95 | 8 | 8 |
| 16 | 4 | 4 | 36 | 4 | 4 | 56 | 7 | 7 | 76 | 7 | 7 | 96 | 9 | 9 |
| 17 | 5 | 5 | 37 | 4 | 4 | 57 | 6 | 6 | 77 | 11 | 11 | 97 | 9 | 9 |
| 18 | 6 | 6 | 38 | 6 | 6 | 58 | 11 | 11 | 78 | 7 | 7 | 98 | 8 | 8 |
| 19 | 4 | 4 | 39 | 4 | 4 | 59 | 12 | 12 | 79 | 12 | 12 | 99 | 6 | 6 |
| 20 | 5 | 5 | 40 | 7 | 7 | 60 | 12 | 12 | 80 | 12 | 12 | 100 | 8 | 8 |

Supplementary Table 10. Vertebra label result record table

Vertebra label result record table, which records the actual number of vertebra (abbreviated as Nact) and the number of vertebra labels correctly identified by algorithm (abbreviated as Ncorr) in each case.

| ID | Nact | Ncorr | ID | Nact | Ncorr | ID | Nact | Ncorr | ID | Nact | Ncorr | ID | Nact | Ncorr |
| --- | --- | --- | --- | --- | --- | --- | --- | --- | --- | --- | --- | --- | --- | --- |
| 1 | 5 | 5 | 21 | 4 | 4 | 41 | 6 | 6 | 61 | 12 | 12 | 81 | 11 | 11 |
| 2 | 8 | 8 | 22 | 5 | 5 | 42 | 7 | 7 | 62 | 11 | 11 | 82 | 6 | 6 |
| 3 | 7 | 7 | 23 | 5 | 5 | 43 | 4 | 4 | 63 | 12 | 12 | 83 | 6 | 6 |
| 4 | 6 | 6 | 24 | 4 | 4 | 44 | 5 | 5 | 64 | 11 | 11 | 84 | 6 | 6 |
| 5 | 9 | 9 | 25 | 5 | 5 | 45 | 7 | 7 | 65 | 9 | 9 | 85 | 8 | 8 |
| 6 | 7 | 7 | 26 | 5 | 5 | 46 | 6 | 6 | 66 | 12 | 12 | 86 | 12 | 12 |
| 7 | 8 | 8 | 27 | 4 | 4 | 47 | 4 | 4 | 67 | 11 | 11 | 87 | 12 | 12 |
| 8 | 5 | 5 | 28 | 6 | 6 | 48 | 5 | 5 | 68 | 9 | 9 | 88 | 8 | 8 |
| 9 | 4 | 4 | 29 | 4 | 4 | 49 | 6 | 6 | 69 | 12 | 12 | 89 | 11 | 11 |
| 10 | 4 | 4 | 30 | 9 | 9 | 50 | 4 | 4 | 70 | 10 | 10 | 90 | 6 | 6 |
| 11 | 4 | 4 | 31 | 4 | 4 | 51 | 7 | 7 | 71 | 11 | 11 | 91 | 7 | 7 |
| 12 | 4 | 4 | 32 | 7 | 7 | 52 | 8 | 8 | 72 | 7 | 7 | 92 | 6 | 6 |
| 13 | 4 | 4 | 33 | 7 | 7 | 53 | 8 | 8 | 73 | 8 | 8 | 93 | 11 | 11 |
| 14 | 4 | 4 | 34 | 7 | 7 | 54 | 7 | 7 | 74 | 8 | 8 | 94 | 10 | 10 |
| 15 | 6 | 6 | 35 | 6 | 6 | 55 | 6 | 6 | 75 | 8 | 8 | 95 | 8 | 8 |
| 16 | 4 | 4 | 36 | 4 | 4 | 56 | 7 | 7 | 76 | 7 | 7 | 96 | 9 | 9 |
| 17 | 5 | 5 | 37 | 5 | 5 | 57 | 6 | 6 | 77 | 11 | 11 | 97 | 9 | 9 |
| 18 | 6 | 6 | 38 | 6 | 6 | 58 | 11 | 11 | 78 | 7 | 7 | 98 | 8 | 8 |
| 19 | 4 | 4 | 39 | 4 | 4 | 59 | 12 | 12 | 79 | 12 | 12 | 99 | 6 | 6 |
| 20 | 5 | 5 | 40 | 7 | 7 | 60 | 12 | 12 | 80 | 12 | 12 | 100 | 8 | 8 |

Supplementary Table 11. Quality factor results record table

Quality factor results record table, which records the results of manual judgment (abbreviated as Manual) and quality factor algorithm (abbreviated as QF)

| ID | Manual | QF | ID | Manual | QF | ID | Manual | QF | ID | Manual | QF |
| --- | --- | --- | --- | --- | --- | --- | --- | --- | --- | --- | --- |
| 1 | qualified | 0.98 | 26 | qualified | 0.95 | 51 | qualified | 0.97 | 76 | qualified | 0.94 |
| 2 | qualified | 0.88 | 27 | qualified | 0.88 | 52 | qualified | 0.94 | 77 | qualified | 0.86 |
| 3 | qualified | 0.88 | 28 | qualified | 0.87 | 53 | qualified | 0.93 | 78 | qualified | 0.92 |
| 4 | qualified | 0.91 | 29 | qualified | 0.87 | 54 | qualified | 0.89 | 79 | qualified | 1.00 |
| 5 | qualified | 0.96 | 30 | qualified | 0.93 | 55 | qualified | 0.87 | 80 | qualified | 0.90 |
| 6 | qualified | 0.89 | 31 | qualified | 0.84 | 56 | qualified | 0.93 | 81 | qualified | 0.90 |
| 7 | qualified | 0.91 | 32 | qualified | 0.82 | 57 | qualified | 0.91 | 82 | unqualified | 0.58 |
| 8 | qualified | 0.86 | 33 | qualified | 0.92 | 58 | qualified | 0.87 | 83 | qualified | 0.98 |
| 9 | unqualified | 1.00 | 34 | qualified | 0.89 | 59 | qualified | 0.85 | 84 | qualified | 0.91 |
| 10 | qualified | 0.81 | 35 | qualified | 0.84 | 60 | qualified | 0.91 | 85 | qualified | 0.92 |
| 11 | qualified | 0.78 | 36 | qualified | 0.83 | 61 | qualified | 0.87 | 86 | qualified | 0.78 |
| 12 | qualified | 0.90 | 37 | qualified | 0.94 | 62 | qualified | 0.97 | 87 | qualified | 0.84 |
| 13 | qualified | 0.97 | 38 | qualified | 0.91 | 63 | qualified | 0.94 | 88 | qualified | 0.91 |
| 14 | qualified | 0.90 | 39 | qualified | 0.87 | 64 | qualified | 0.97 | 89 | qualified | 0.92 |
| 15 | qualified | 0.91 | 40 | qualified | 0.92 | 65 | qualified | 0.93 | 90 | qualified | 0.93 |
| 16 | qualified | 0.86 | 41 | qualified | 0.89 | 66 | qualified | 0.96 | 91 | qualified | 0.80 |
| 17 | qualified | 0.87 | 42 | qualified | 0.95 | 67 | qualified | 0.95 | 92 | qualified | 0.95 |
| 18 | qualified | 0.91 | 43 | qualified | 0.84 | 68 | qualified | 0.98 | 93 | qualified | 0.97 |
| 19 | unqualified | 0.83 | 44 | qualified | 0.79 | 69 | qualified | 0.93 | 94 | qualified | 0.99 |
| 20 | qualified | 0.85 | 45 | qualified | 0.89 | 70 | qualified | 0.90 | 95 | qualified | 0.95 |
| 21 | qualified | 0.77 | 46 | qualified | 0.88 | 71 | qualified | 0.97 | 96 | qualified | 0.99 |
| 22 | qualified | 0.84 | 47 | qualified | 0.94 | 72 | qualified | 1.00 | 97 | qualified | 0.93 |
| 23 | qualified | 0.95 | 48 | qualified | 0.90 | 73 | qualified | 0.98 | 98 | unqualified | 0.62 |
| 24 | qualified | 0.93 | 49 | qualified | 0.94 | 74 | qualified | 0.94 | 99 | qualified | 0.98 |
| 25 | qualified | 0.78 | 50 | qualified | 0.79 | 75 | qualified | 0.86 | 100 | qualified | 0.94 |

Supplementary Table 12. The results of Bland-Altman methods.

This table shows all the results of the BA detection method. Contains 95%CI and SD. (OA: Offset angle; PA: Psoas area; FJA: Facet joint angle; DH: Disc height; LLRVD: Left lateral foramen vertical diameter; RLFVD: Right lateral foramen vertical diameter; SCD: Spinal canal diameter; LLRH: Left lateral recess height; RLRH: Right lateral recess height; LLRA: Left lateral recess angle; RLRA: Right lateral recess angle; LDHR: Lumbar disc herniation ratio) DL: deep learning group, HM: human-machine group, FM: fully manual group.

|  | DL VS HM | | DL VS FM | | HM VS FM | |
| --- | --- | --- | --- | --- | --- | --- |
| OA | Bias 95% CI | SD | Bias 95% CI | SD | Bias 95% CI | SD |
| L1/2 | -0.4809(-0.4809, 0.8501) | 0.8501 | -1.015(-3.515, 1.486) | 1.276 | -0.6000(-3.137, 1.937) | 1.249 |
| L2/3 | -0.2754(-2.074, 1.523) | 0.9175 | -0.7250(-3.148, 1.698) | 1.236 | -0.5426(-2.576, 1.490) | 1.037 |
| L3/4 | -0.2548(-2.405, 1.895) | 1.097 | -0.7365(-3.281, 1.810) | 1.299 | -0.4604(-3.097, 2.176) | 1.345 |
| L4/5 | 0.05814(-1.994, 2.110) | 1.047 | -0.4729(-3.360, 2.414) | 1.473 | -0.4607(-3.057, 2.135) | 1.325 |
| L5/1 | -0.3133(-3.577, 2.950) | 1.665 | -1.079(-5.605, 3.447) | 2.309 | -0.5132(-4.454, 3.427) | 2.010 |
| PA | Bias 95% CI | SD | Bias 95% CI | SD | Bias 95% CI | SD |
| L1/2 | 0.01250(-0.5058, 0.5308) | 0.2644 | -0.1240(-0.7822, 0.5342) | 0.3358 | -0.1598(-0.7818, 0.4621) | 0.3173 |
| L2/3 | -0.02224(-1.176, 1.132) | 0.5887 | -0.1292(-1.372, 1.114) | 0.6343 | -0.1347(-1.345, 1.076) | 0.6175 |
| L3/4 | 0.1058(-1.352, 1.564) | 0.7438 | -0.08646(-1.708, 1.535) | 0.8273 | -0.2127(-1.749, 1.324) | 0.7838 |
| L4/5 | 0.05654(-1.713, 1.826) | 0.9029 | -0.1931(-2.191, 1.804) | 1.019 | -0.1589(-2.091, 1.773) | 0.9857 |
| L5/1 | 0.1636(-1.802, 2.129) | 1.003 | -0.1826(-2.231, 1.866) | 1.045 | -0.3230(-2.401, 1.755) | 1.060 |
| FJA | Bias 95% CI | SD | Bias 95% CI | SD | Bias 95% CI | SD |
| L1/2 | 0.4932(-11.39, 12.37) | 6.062 | -1.653(-13.62, 10.32) | 6.107 | -1.097(-15.10, 12.91) | 7.145 |
| L2/3 | 0.2369(-12.09, 12.57) | 6.291 | -1.448(-13.71, 10.82) | 6.258 | -1.001(-13.69, 11.69) | 6.476 |
| L3/4 | 0.7827(-10.68, 12.25) | 5.849 | -1.408(-14.86, 12.05) | 6.865 | -0.7159(-14.34, 12.91) | 6.951 |
| L4/5 | 1.062(-11.04, 13.17) | 6.176 | -1.672(-15.32, 11.97) | 6.962 | -1.208(-13.74, 11.33) | 6.395 |
| L5/1 | 1.410(-9.792, 12.61) | 5.716 | -1.352(-15.28, 12.58) | 7.107 | -0.9254(-14.21, 12.36) | 6.776 |
| DH | Bias 95% CI | SD | Bias 95% CI | SD | Bias 95% CI | SD |
| L1/2 | 0.06250(-0.3908, 0.5158) | 0.2313 | -0.1091(-0.5288, 0.3106) | 0.2141 | -0.1596(-0.5910, 0.2717) | 0.2201 |
| L2/3 | 0.07250(-0.4029, 0.5479) | 0.2425 | -0.1053(-0.5806, 0.3700) | 0.2425 | -0.1591(-0.6515, 0.3332) | 0.2512 |
| L3/4 | 0.05424(-0.4526, 0.5611) | 0.2586 | -0.1025(-0.6211, 0.4161) | 0.2646 | -0.1441(-0.6536, 0.3654) | 0.2600 |
| L4/5 | 0.03464(-0.5720, 0.6413) | 0.3095 | -0.08659(-0.6737, 0.5005) | 0.2995 | -0.1572(-0.7681, 0.4627) | 0.3140 |
| L5/1 | 0.01800(-0.7251, 0.7611) | 0.3791 | -0.07556(-0.7407, 0.5896) | 0.3394 | -0.1355(-0.7407, 0.4697) | 0.3088 |
| LLFVD | Bias 95% CI | SD | Bias 95% CI | SD | Bias 95% CI | SD |
| L1/2 | -0.04312(-0.6370, 0.5508) | 0.3030 | -0.01030(-0.5995, 0.5789) | 0.3006 | 0.0004412(-0.5709, 0.5718) | 0.2915 |
| L2/3 | -0.06635(-0.8324, 0.6997) | 0.3908 | -0.05475(-0.7794, 0.6699) | 0.3697 | -0.01292(-0.7479, 0.7220) | 0.3750 |
| L3/4 | -0.1029(-0.9268, 0.7209) | 0.4203 | -0.08208(-0.8205, 0.6563) | 0.3767 | -0.04971(-0.7460, 0.6466) | 0.3552 |
| L4/5 | -0.1001(-0.8449, 0.6446) | 0.3800 | -0.03446(-0.8025, 0.7335) | 0.3918 | -0.02203(-0.6869, 0.6429) | 0.3392 |
| L5/1 | 0.001071(-0.6638, 0.6659) | 0.3392 | -0.02964(-0.6931, 0.6338) | 0.3385 | -0.1096(-0.7592, 0.5399) | 0.3314 |
| RLFVD | Bias 95% CI | SD | Bias 95% CI | SD | Bias 95% CI | SD |
| L1/2 | -0.05345(-0.6407, 0.5338) | 0.2996 | -0.02885(-0.6115, 0.5538) | 0.2973 | -0.02396(-0.5843, 0.5364) | 0.2859 |
| L2/3 | -0.08847(-0.8820, 0.7051) | 0.4049 | -0.06914(-0.8129, 0.6746) | 0.3795 | -0.07489(-0.8175, 0.6677) | 0.3789 |
| L3/4 | -0.1399(-0.9022, 0.6225) | 0.3890 | -0.1135(-0.8369, 0.6099) | 0.3691 | -0.04713(-0.7499, 0.6557) | 0.3586 |
| L4/5 | -0.09638(-0.8268, 0.6341) | 0.3727 | -0.07157(-0.8098, 0.6667) | 0.3767 | -0.06101(-0.7644, 0.6424) | 0.3589 |
| L5/1 | -0.03956(-0.6678, 0.5887) | 0.3205 | -0.04806(-0.6762, 0.5801) | 0.3205 | -0.05619(-0.6590, 0.5466) | 0.3075 |
| SCD | Bias 95% CI | SD | Bias 95% CI | SD | Bias 95% CI | SD |
| L1/2 | 0.1067 (-0.4457, 0.6591) | 0.2818 | 0.03523 (-0.5797, 0.6502) | 0.3138 | -0.09957 (-0.6832, 0.4841) | 0.2978 |
| L2/3 | 0.1243 (-0.4958, 0.7443) | 0.3164 | 0.001612 (-0.6852, 0.6884) | 0.3504 | -0.1457 (-0.7926, 0.5013) | 0.3301 |
| L3/4 | 0.1112 (-0.6169, 0.8394) | 0.3715 | -0.03783 (-0.8437, 0.7680) | 0.4112 | -0.1602 (-0.9234, 0.6029) | 0.3894 |
| L4/5 | 0.07357 (-0.6651, 0.8122) | 0.3769 | -0.03471 (-0.9330, 0.8635) | 0.4583 | -0.1540 (-1.004, 0.6955) | 0.4334 |
| L5/1 | 0.1008 (-0.6265, 0.8281) | 0.3711 | 0.05049 (-0.7623, 0.8633) | 0.4147 | -0.09827 (-0.8716, 0.6751) | 0.3946 |
| LLRH | Bias 95% CI | SD | Bias 95% CI | SD | Bias 95% CI | SD |
| L1/2 | 0.1564 (-3.833, 4.146) | 2.036 | -0.5437 (-4.607, 3.520) | 2.073 | -0.5234 (-4.332, 3.285) | 1.943 |
| L2/3 | -0.02457 (-4.295,4.246) | 2.179 | -0.5835 (-4.650, 3.483) | 2.075 | -0.3224 (-4.515, 3.871) | 2.139 |
| L3/4 | -0.05907 (-3.750, 3.632) | 1.883 | -0.6844 (-4.342, 2.973) | 1.866 | -0.5849 (-4.393, 3.223) | 1.943 |
| L4/5 | 0.02664 (-2.995, 3.048) | 1.542 | -0.2316 (-3.616, 3.153) | 1.727 | -0.4201 (-3.684, 2.844) | 1.665 |
| L5/1 | 0.4289 (-3.741, 4.598) | 2.127 | 0.01588 (-4.084, 4.116) | 2.092 | -0.4122 (-4.443, 3.619) | 2.057 |
| RLRH | Bias 95% CI | SD | Bias 95% CI | SD | Bias 95% CI | SD |
| L1/2 | 0.3254 (-4.004, 4.654) | 2.209 | -0.3807 (-4.437, 3.676) | 2.070 | -0.5817 (-4.733, 3.569) | 2.118 |
| L2/3 | 0.3636 (-4.087, 4.814) | 2.271 | -0.5695 (-4.705, 3.566) | 2.110 | -0.6477 (-4.476, 3.181) | 1.953 |
| L3/4 | 0.07829 (-3.693, 3.850) | 1.924 | -0.7227 (-4.647, 3.202) | 2.002 | -0.6446 (-4.349, 3.060) | 1.890 |
| L4/5 | 0.05979 (-3.129, 3.248) | 1.627 | -0.2721 (-3.810, 3.266) | 1.805 | -0.4780 (-3.450, 2.494) | 1.516 |
| L5/1 | 0.5261 (-3.514, 4.566) | 2.061 | -0.08369 (-4.256, 4.089) | 2.129 | -0.5406 (-4.482, 3.401) | 2.011 |
| LLRA | Bias 95% CI | SD | Bias 95% CI | SD | Bias 95% CI | SD |
| L1/2 | 0.6173 (-13.54, 14.78) | 7.225 | -1.477 (-13.57, 10.62) | 6.171 | -1.172 (-15.41, 13.07) | 7.266 |
| L2/3 | 1.033 (-24.40, 26.47) | 12.98 | -4.133 (-24.04, 15.77) | 10.15 | -4.661 (-25.25, 15.93) | 10.51 |
| L3/4 | 0.3021 (-27.09, 27.70) | 13.98 | -5.405 (-30.13, 19.32) | 12.62 | -5.991 (-31.08, 19.10) | 12.80 |
| L4/5 | 1.703 (-23.40, 26.80) | 12.81 | -3.830 (-29.98, 22.31) | 13.34 | -5.018 (-30.65, 20.62) | 13.08 |
| L5/1 | 3.481 (-21.48, 28.44) | 12.73 | -0.8666 (-27.31, 25.58) | 13.49 | -5.313 (-31.29, 20.66) | 13.25 |
| RLRA | Bias 95% CI | SD | Bias 95% CI | SD | Bias 95% CI | SD |
| L1/2 | 1.180 (-9.383, 11.74) | 5.389 | 0.09400 (-12.11, 12.30) | 6.227 | -1.605 (-13.06, 9.851) | 5.845 |
| L2/3 | 2.880 (-20.41, 26.17) | 11.88 | -1.339 (-20.09, 17.41) | 9.568 | -3.415 (-23.05, 16.22) | 10.02 |
| L3/4 | 1.836 (-25.20, 28.87) | 13.79 | -4.119 (-32.33, 24.09) | 14.39 | -6.242 (-30.28, 17.79) | 12.26 |
| L4/5 | 1.982 (-21.87, 25.83) | 12.17 | -4.070 (-32.14, 24.00) | 14.32 | -5.074 (-28.88, 18.73) | 12.14 |
| L5/1 | 4.090 (-20.98, 29.16) | 12.79 | -1.856 (-28.95, 25.24) | 13.82 | -6.076 (-31.55, 19.40) | 13.00 |
| LDHR | Bias 95% CI | SD | Bias 95% CI | SD | Bias 95% CI | SD |
| L1/2 | -0.007220 (-0.1850, 0.1706) | 0.09071 | -0.003157(-0.1454, 0.1391) | 0.07257 | 0.009419 (-0.1578, 0.1767) | 0.08533 |
| L2/3 | -0.03303 (-0.3460, 0.2800) | 0.1597 | 0.01749 (-0.2682, 0.3031) | 0.1457 | 0.03577 (-0.2640, 0.3356) | 0.1530 |
| L3/4 | -0.01817 (-0.4714, 0.4351) | 0.2312 | 0.05308 (-0.3557, 0.4619) | 0.2086 | 0.07247 (-0.3472, 0.4921) | 0.2141 |
| L4/5 | 0.007052 (-0.4465, 0.4606) | 0.2314 | 0.02137 (-0.4844, 0.5272) | 0.2581 | 0.03292 (-0.4581, 0.5240) | 0.2505 |
| L5/1 | -0.02690 (-0.4015, 0.3477) | 0.1911 | -0.01919 (-0.4703, 0.4319) | 0.2302 | -0.003987 (-0.4171, 0.4091) | 0.2108 |

Supplementary Table 13. Network Configuration of the LVP System.

Network configurations and output definitions of the three-stage V-Net models in the LVP framework.

| Network Type | Task | Input | Output | Number of Parameters |
| --- | --- | --- | --- | --- |
| V-Net-1 | Spine localization | 3D volumetric data | Probability map of spine mask segmentation | 55.6 MB |
| V-Net-2 | Intervertebral disc segmentation | 3D image patches cropped according to the localized spine mask | Probability map of intervertebral disc segmentation | 55.6 MB |
| V-Net-3 | Intervertebral disc labeling (point localization) | 3D image patches cropped according to the localized spine mask | 23-channel heatmap corresponding to 23 intervertebral discs, where each channel represents the probability of the disc center position | 55.8 MB |

Supplementary Figure 1-5

Supplementary Figure 1. Performance verification of segmentation models constructed based on V-Net and nnU-Net. Comparison of segmentation performance between V-Net and nnU-Net across 149 paired cases. Boxes show the median and interquartile range; whiskers indicate 1.5 × IQR. No statistically significant difference was observed (Wilcoxon signed-rank test, p = 0.389).


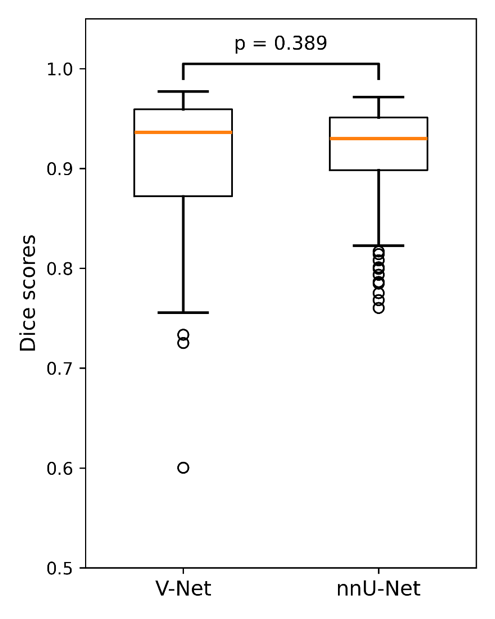


Supplementary Figure 2. Positioning of different positioning lines

Accurate description of sagittal and coronal positioning lines. DL and HM had better position effect. DL: deep learning group, HM: human-machine group, FM: fully manual group.


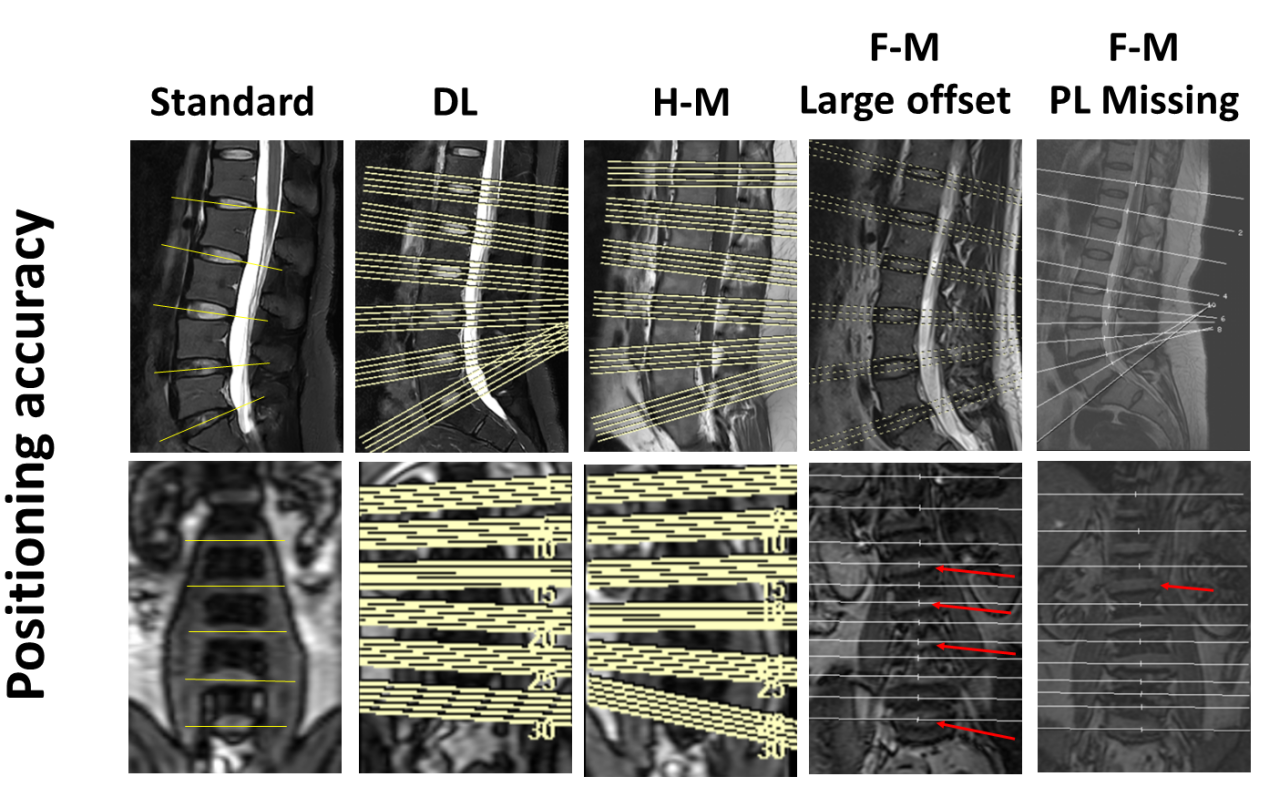


Supplementary Figure 3. Lumbar annotation procedure.

The annotation process involved three steps: spine segmentation, intervertebral disc segmentation, and disc labeling. First, the spine mask was outlined to fully cover vertebrae and discs. Next, disc masks were segmented to define their shapes, followed by precise labeling of disc centers. Quality control criteria included: precise spinal contour alignment, accurate disc morphology representation, and anatomically correct label placement.


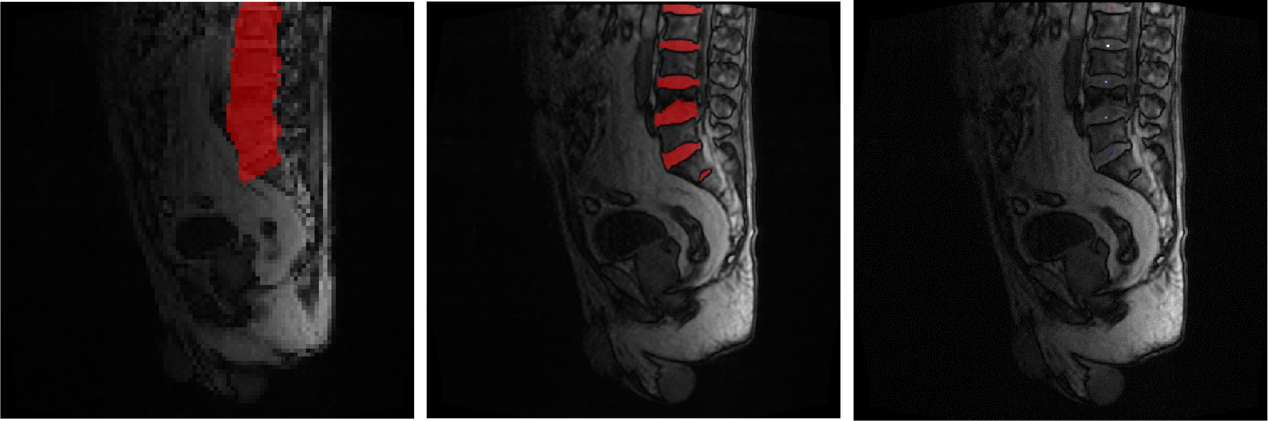


Supplementary Figure 4. The AI model development items.

Specific parameters for model development include nine items: best displaying sagittal image, best displaying coronal image, sagittal scanning center and direction, coronal scanning center and direction, transverse scanning center and direction, saturation band, disc/vertebral label and quality factor.


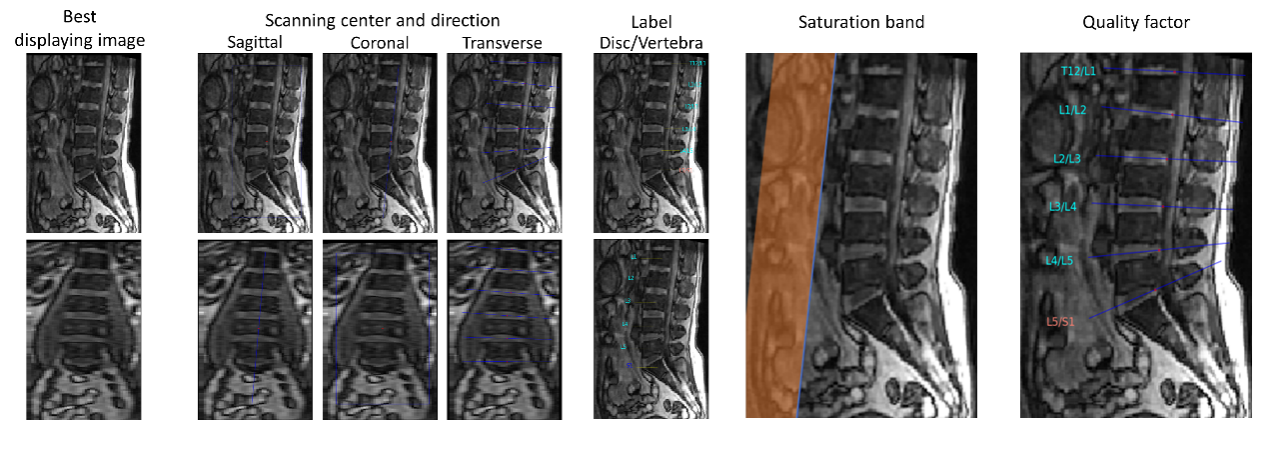


Supplementary Figure 5. The Matching error of Intervertebral Disc Label Module.


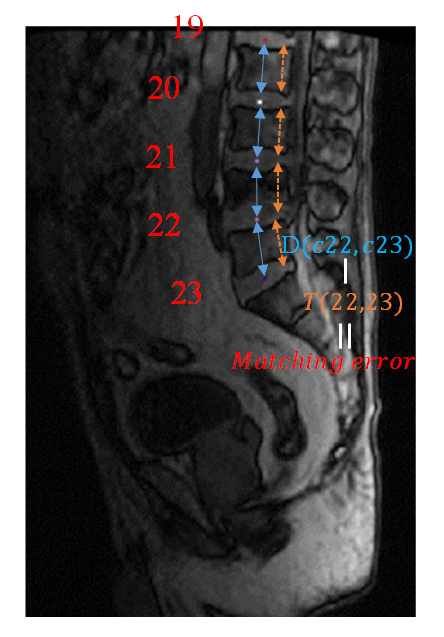


Supplementary Movie 1

Supplementary Movie 1: Movie comparison of LVP system and manual operation

This supplementary movie provides a comparison between the LVP system and a full manual MRI. LVP systems offer faster speeds and easier operation processes. It is convenient for clinical application to a great extent. Below are two links to this video (both links correspond to the same video, just choose one of them).

<https://drive.google.com/file/d/1TNj1bxTXtUSh1eZlqNz9smQCa-Zd5ZDy/view?usp=sharing>

Figures S1 to S5

Tables S1 to S13

Movies S1
